# Polysaccharide Adjuvants as Innate Immune Trainers: Bridging Pattern Recognition Receptor (PRR) Activation and Metabolic Reprogramming for Synthetic Vaccine Design

**DOI:** 10.1002/advs.202509022

**Published:** 2025-11-25

**Authors:** Jeong Hyun Moon, May Thazin Phoo, Yubin Kim, Jinsung Ahn, April Kim, Jutaek Nam, Soo‐Hong Lee, James J. Moon, Sejin Son

**Affiliations:** ^1^ Department of Biological Sciences and Bioengineering, Inha University/Industry‐Academia, Interactive R&E Center for Bioprocess Innovation Inha University Incheon 22212 Republic of Korea; ^2^ Department of Pharmaceutical Sciences University of Michigan Ann Arbor MI 48109 USA; ^3^ Department of Biomedical Engineering Dongguk University Seoul 04620 Republic of Korea; ^4^ Biointerfaces Institute University of Michigan Ann Arbor MI 48109 USA; ^5^ College of Pharmacy Chonnam National University Gwangju 61186 Republic of Korea; ^6^ Department of Biomedical Engineering University of Michigan Ann Arbor MI 48109 USA; ^7^ Department of Chemical Engineering University of Michigan Ann Arbor MI 48109 USA; ^8^ Department of Biological Sciences Inha University Incheon 22212 Republic of Korea

**Keywords:** immune engineering, immunoadjuvant, PAMP‐PRR, polysaccharide, trained immunity

## Abstract

The concept of trained immunity has redefined the understanding of innate immune memory and opened new opportunities for vaccine design. Polysaccharides, as naturally occurring pathogen‐associated molecular patterns (PAMPs), can activate pattern recognition receptors (PRRs) and induce durable immunomodulatory effects. This review examines the historical context of microbial immunotherapy, beginning with Coley's toxin, and traces its evolution toward the rational use of polysaccharides as vaccine adjuvants. Their mechanisms of action, ranging from PRR engagement to metabolic and epigenetic reprogramming, are discussed to support both innate training and adaptive immune activation. Emphasis is placed on how these materials interact with biological barriers, influence antigen processing, and enhance lymph node trafficking. By analyzing the immunological functions and material properties of β‐glucan, mannan, alginate, hyaluronic acid, chitosan, and others, the potential of polysaccharide‐based platforms is highlighted to improve the efficacy and breadth of synthetic vaccines.

## Graphical abstract

Polysaccharides modulate immune responses by engaging pattern recognition receptors (PRRs) to induce T‐cell activation. In vaccine design, their particle size critically influences lymph node targeting and activation mechanisms. By engineering structural complexity and multivalent PRR engagement, polysaccharides enable precise modulation of immune signaling, offering versatile potential for diverse biomedical applications.

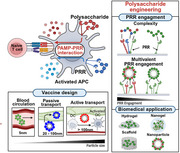

## 1 Introduction

### 1.1 Coley's Toxin

Coley's Toxin is a foundational milestone in cancer immunotherapy, having paved the way for modern treatments by showing that stimulating the immune system can be effective against certain cancers.^[^ ^1^ ^]^ Coley's Toxin, developed by William Coley, was an early form of cancer immunotherapy using heat‐killed bacteria to treat osteosarcoma. Inspired by a patient's tumor regression following a streptococcal infection, Coley hypothesized that bacterial infections could stimulate antitumor immunity.^[^ ^2^, ^3^ ^]^ Despite Coley's reports of significant success with his toxins, many of his colleagues responded with severe criticisms towards his finding due to variation in toxin preparation, inconsistent methods of administrations, inadequately controlled and documented patient follow‐ups, absence of safety and efficacy data, and a limited understanding of the scientific rationale behind cancer treatment, leading to its disapproval for clinical use.^[^ ^1^, ^2^ ^]^ However, reevaluation of his toxins years after his passing revealed the induction of antitumor effects,^[^ ^4^ ^]^ and his work encouraged further investigation of microbe‐derived therapeutics for enhancing innate immunity. One notable instance is the approval of Bacillus Calmette‐Guérin (BCG) vaccine, discovered to elicit trained immunity, for treatment of highrisk non‐muscle‐invasive bladder cancer.^[^ ^5^ ^]^ This pioneering work signifies the initiation of contemporary cancer immunotherapy approaches. The discovery of pattern recognition receptors (PRRs) provides a strong rationale for the use of Coley's Toxin and elucidates why bacterial infections can trigger anti‐cancer immune responses.

### 1.2 Immune Response to Bacteria (Pathogen) Infection

The human body has developed multiple defense mechanisms to manage resident microbes and combat pathogens, including physical barriers (skin and mucosa), mechanical defenses (tight junctions and cilia), and biochemical barriers (antimicrobial enzymes in tears and saliva). In efforts to maintain the body's defense and overall health, the immune system employs two interdependent responses: the innate and adaptive immune responses. These responses serve to protect the host from foreign substances and pathogens. The innate immune system, recognized as the body's first line of defense, responds to a rapid and nonspecific response by recognizing pathogen through PRRs that detect pathogen associated molecular pattern (PAMP) or damage‐associated molecular pattern (DAMP).^[^ ^6^ ^]^ The recognition of a pathogen by the innate immune system at the cell membrane depends on various factors such as the host cell type, expressed membrane receptors, pathogen itself, and specific conditions of host‐pathogen interaction.^[^ ^7^ ^]^ The collective activation of various PRRs, such as toll‐like receptors (TLRs), as well as complement receptors, fragment crystallizable (Fc) gamma receptors, retinoic acid‐inducible gene‐I (RIG‐I)‐like receptors (RLRs), and nucleotide‐binding oligomerization domain and leucine‐rich repeat‐containing receptors (NLRs), is crucial for initiating an effective innate immune response.^[^ ^7^, ^8^, ^9^ ^]^

## 2 Emergence of Synthetic Vaccine

### 2.1 Definition and Remarkable Success of SARS‐CoV‐2 virus (Covid‐19) Synthetic Vaccine in the Beginning

Vaccines, biological agents designed to elicit immune responses for protection against infections or diseases upon pathogen exposure, essentially contain protein antigens sourced from pathogens or synthetic components mimicking pathogen antigens.^[^ ^10^ ^]^ In contrast to traditional whole‐pathogen vaccines, subunit vaccines, which use only specific parts of a pathogen, offer advantages in terms of safety and precision; however, necessitating the use of adjuvants for optimal vaccine efficacy due to low immunogenicity.^[^ ^11^ ^]^ In the domain of vaccines, an adjuvant is described as a component capable of stimulating innate immune cells to increase and/or modulate the intensity and duration of antigen‐specific immune responses.^[^ ^12^, ^13^ ^]^ Highly effective vaccines possess the capability to induce sustained production of neutralizing antibodies (humoral immunity) and cell‐mediated immunity, thus offering durable, protection against disease.^[^ ^14^ ^]^

The recent widespread administration mRNA vaccines for SARS‐CoV‐2 virus (Covid‐19) highlights an innovative biomedical technology.^[^ ^38^, ^39^ ^]^ The application of mRNA technology provides numerous benefits compared to traditional approaches, such as accelerated manufacturing timelines and a versatile design through simple modifications to the mRNA sequence.^[^ ^40^ ^]^ Structural modifications of mRNA in Covid‐19 vaccines demonstrated increased dose tolerance, rapid antibody response, and higher translational efficiency.^[^ ^15^ ^]^ This vaccine opened a new era of synthetic vaccines.^[^ ^16^ ^]^

### 2.2 The Generation of Immune Response to Vaccine

The initial encounter between the vaccine and the immune system at the injection site is pivotal, encompassing the infiltration, activation, and antigen uptake of innate immune cells, processes that shape the quality of the adaptive response.^[^ ^17^, ^18^ ^]^ Antigen‐presenting cells (APCs) uptake antigens and process them into peptides, which are then presented via major histocompatibility complex (MHC) molecules presentation (signal 1).^[^ ^19^ ^]^ Many immunostimulatory adjuvants interact with PRRs on innate immune cells, promoting the release of cytokines (e.g., Interleukin (IL) ‐6, IL‐10, IL‐12, tumor necrosis factor (TNF)‐α, and interferon (IFN)). This recruits additional immune cells, enhances inflammation (Signal 3), and induces co‐stimulatory molecules (signal 2) like CD40, CD80, and CD86 on APCs, which are essential for effective T cell activation.^[^ ^14^, ^20^ ^]^

Activated dendritic cells (DCs) and vaccine components migrate via to the draining lymph nodes,^[^ ^21^, ^22^ ^]^ where they organize spatially to facilitate effective cell‐to‐cell interactions for a robust immune response.^[^ ^22^, ^23^ ^]^ Lymph node resident cells activate naïve CD8^+^ T cells through peptide‐MHC I complexes, T‐cell receptor (TCR) engagement, co‐stimulatory molecules, and cytokines, leading to the expansion of antigen‐specific CD8^+^ T cells^[^ ^23^, ^24^ ^]^ and the generation of memory subsets for long‐term protection.^[^ ^25^, ^26^ ^]^ Meanwhile, APCs also induce humoral immunity by presenting antigens to both CD4^+^ T cells and B cells, promoting B cell maturation, class switching, and antibody affinity enhancement.^[^ ^14^, ^27^, ^28^ ^]^

**Figure 1:**
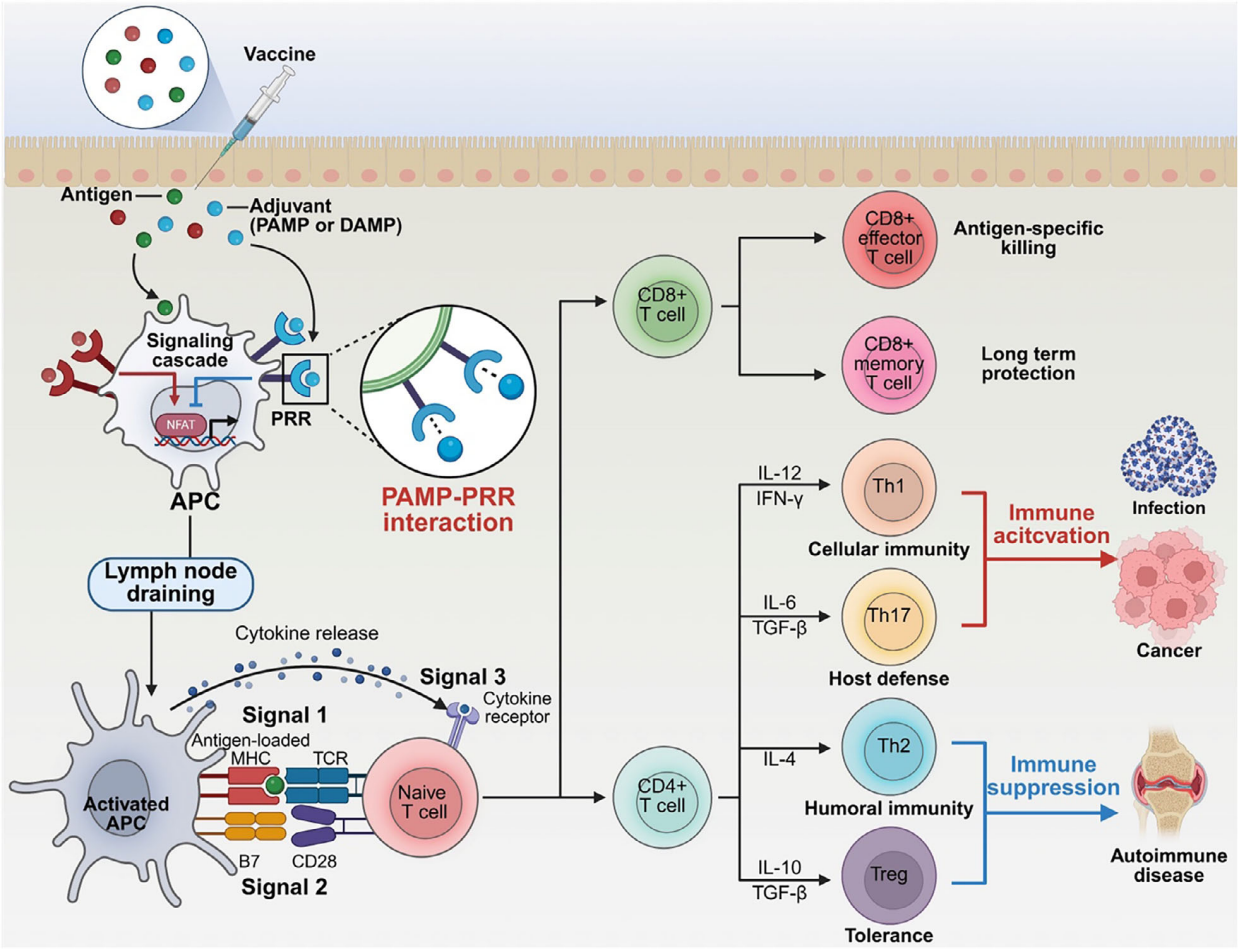
Through the recognition of pathogen‐associated molecular patterns (PAMPs) by pattern recognition receptors (PRRs) (PAMP‐PRR interaction), vaccines enable to trigger specific immune responses that contribute to their therapeutic efficacy. Vaccines are generally composed of two main components: antigens and adjuvants. Following administration, antigen‐presenting cells (APCs) internalize the antigen, while adjuvants activate innate immune pathways by engaging PRRs through PAMP recognition (PAMP‐PRR interaction). This activation enhances lymphatic drainage toward the lymph nodes, where priming of adaptive immune responses occurs. Activated APCs interact with naïve T cells through three essential signals: Signal 1, the presentation of antigenic peptides via major histocompatibility complex (MHC) molecules to T cell receptors (TCRs); Signal 2, the expression of co‐stimulatory molecules; and Signal 3, the secretion of cytokines for T cell differentiation. T cells become activated and differentiate into either CD8⁺ or CD4⁺ subsets. CD8⁺ T cells expand into cytotoxic effector cells for antigen‐specific target cell elimination and differentiate to memory T cells for long‐term immune protection. CD4⁺ T cells, influenced by the cytokine environment, differentiate into various functional subsets. Th1 and Th17 cells are involved in promoting immune responses against infection and cancer, whereas Th2 and regulatory T cell (Treg) subsets contribute to the regulation and suppression of immune activity, providing therapeutic potential for autoimmune diseases.

Naive CD4⁺ T cells differentiate into specialized subsets of effector CD4⁺ T helper (TH) cells, critically contributing to the establishment of a distinct cytokine environment that orchestrates specific adaptive immune responses.^[^ ^29^, ^30^ ^]^ Following differentiation, each CD4^+^ T cell subset acquires distinct immunological functions. Th1 cells are induced by IL‐12 and IFN‐γ and promote cellular immunity primarily through the activation of CD8^+^ T cells and natural killer (NK) cells. In contrast, Th2 cells differentiate in response to IL‐4 and are mainly involved in humoral immune responses, with significant implications in allergic diseases.^[^ ^31^ ^]^ Th17 cells, driven by transforming growth factor‐beta (TGF‐β) and IL‐6, play a crucial role in host defense against extracellular pathogens by mediating protective inflammatory responses.^[^ ^32^ ^]^ Regulatory T cells (Tregs), induced by IL‐10 and TGF‐β, are essential for maintaining immune tolerance to self‐antigens and suppressing excessive immune activation. Broadly, Th1 and Th17 subsets contribute to immune activation, whereas Th2 and Treg subsets are associated with immune suppression. Modulating these subsets offers therapeutic potential: enhancing immune activation may be beneficial in treating cancer and infectious diseases, while promoting immune suppression could be advantageous in controlling autoimmune disorders (**Figure** **1**).^[^ ^33^ ^]^

## 3 Polysaccharides as a Promising Adjuvant for Vaccine Development

### 3.1 Polysaccharides as a Promising Immune Modulator

A relatively new field of study on immunomodulation using polysaccharides as immunoadjuvants suggested that polysaccharides have significant impacts on the regulation of immune responses. Polysaccharides are a subset of naturally occurring polymers formed by the linkage of carbohydrate monomers through glycosidic bonds (Figure 3a).^[^ ^34^ ^]^ Bacterial cell walls are mainly composed of glycoconjugates and polysaccharides,^[^ ^35^ ^]^ making polysaccharides one of the main initiators of host immunity against infection. Initially, polysaccharides, particularly those of bacterial origin, are believed to only stimulate transient humoral immunity. However, emerging evidence suggests that microbial polysaccharides act as robust immunomodulators, demonstrating their influence on T cells and APCs.^[^ ^36^ ^]^ Incorporation of polysaccharide‐based adjuvants can amplify vaccine through activation of immune cells such as macrophages, T cells, and B cells, and enhanced secretion of molecules such as cytokines, antibodies, and complement molecules. Polysaccharides such as mannan, glucan, alginate, pectin, chitosan, and hyaluronic acid can modulate anti‐inflammatory and pro‐inflammatory cytokines such as IL‐1,^[^ ^37^ ^]^ IL‐2,^[^ ^38^, ^39^ ^]^ IL‐4,^[^ ^39^ ^]^ IL‐1b,^[^ ^40^, ^41^, ^42^ ^]^ IL‐6,^[^ ^37^ ^]^ IL‐9, IL‐10,^[^ ^43^ ^]^ IL‐12,^[^ ^37^ ^]^ IL‐17,^[^ ^44^ ^]^ matrix metalloproteinase‐9 (MMP‐9),^[^ ^40^, ^41^, ^42^ ^]^ IFN‐γ,^[^ ^38^, ^44^ ^]^ and TNF‐α.^[^ ^37^, ^45^, ^46^, ^47^ ^]^ These polysaccharides further contribute to immune modulation through the proliferation of APCs, activation of CD4^+^ and CD8^+^ T cell responses,^[^ ^3^ ^]^ promotion of nitric oxide (NO),^[^ ^48^ ^]^ and/or polarization of macrophage.^[^ ^43^, ^49^ ^]^ Generally, polysaccharides have shown great promise as a field of exploration for therapeutic options in areas such as cancer,^[^ ^50^ ^]^ cardiovascular disease,^[^ ^51^ ^]^ inflammation and infectious disease,^[^ ^52^, ^53^ ^]^ and tissue regeneration.^[^ ^54^ ^]^ Numerous natural and synthetic polysaccharides possess advantageous properties such as low toxicity and excellent biocompatibility and biodegradability.^[^ ^34^ ^]^ In this review paper, we will delve into immunomodulating properties of polysaccharides and their potential as immunoadjuvants.

### 3.2 Pathogen‐Associated Molecular Patterns (PAMP)‐Pattern Recognition Receptors (PRR) Mechanism in Innate Immune Cell

In efforts to maintain the body's defense and overall health, the immune system employs two interdependent responses: the innate and adaptive immune responses. These responses serve to protect the host from foreign substances and pathogens. To do so, innate immune system initiates early innate immune response upon recognition of the pathogens, such as PAMP or DAMP via PRRs expressed on immune cells.^[^ ^6^, ^55^ ^]^ Concisely, PRRs are located in various subcellular compartments, including the cellular and endosomal membranes and the cytosol; the secreted forms of PRRs can also be found extracellularly in the bloodstream and interstitial fluids.^[^ ^56^ ^]^ PRRs can be categorized into four major sub‐families – the TLRs, the NLR, the RLR, and the C‐type lectin receptors (CLRs).^[^ ^57^ ^]^ Among PRRs, TLRs and CLRs are mainly membrane‐bound, with most TLRs (TLR1, TLR2, TLR4, TLR5, and TLR6) expressed on the plasma membrane, whereas others (TLR3, TLR7, TLR8, and TLR9) are found in endosomal compartments, while NLRs and RLRs act as cytosolic sensors. Among them, TLRs and CLRs are primarily known for interacting with polysaccharides. This activation of PRRs subsequently triggers the adaptive immune system through a wide range of pathways and mechanisms to eliminate pathogens.^[^ ^55^, ^58^, ^59^ ^]^

#### 3.2.1 Targeting Surface PRR Pathways

##### TLRs

TLRs, crucial components in the first line defense against pathogens, are classified as type I transmembrane receptors, functioning as the PRRs. These receptors feature short tandem leucine‐rich repeat (LRR) motifs that provide specificity for TLRs in recognizing a diverse range of PAMPs such as lipids, carbohydrates, peptides, and nucleic acids derived from various microorganisms such as bacteria, protozoa, fungi, and viruses.^[^ ^45^, ^60^, ^61^ ^]^ Predominantly, an adaptor protein, Myeloid differentiation primary response 88 (MyD88) mediates TLR signaling^[^ ^62^, ^63^ ^]^ although MyD88‐independent pathways also enables some TLR signaling.^[^ ^63^, ^64^, ^65^ ^]^ These signaling results in gene transcriptions mediated by IFN β‐ and/or nuclear factor‐kappa B (NF‐kB).^[^ ^60^, ^66^, ^67^ ^]^ Such genes include those encoding for MH molecules,^[^ ^68^ ^]^ co‐stimulatory molecules,^[^ ^68^ ^]^ cytokines, chemokines, and adhesion molecules,^[^ ^69^ ^]^ subsequently resulting in mediation of inflammatory response, innate and adaptive immunity coordination,^[^ ^63^, ^70^ ^]^ and elimination of pathogens.^[^ ^62^, ^63^ ^]^

**Figure 2:**
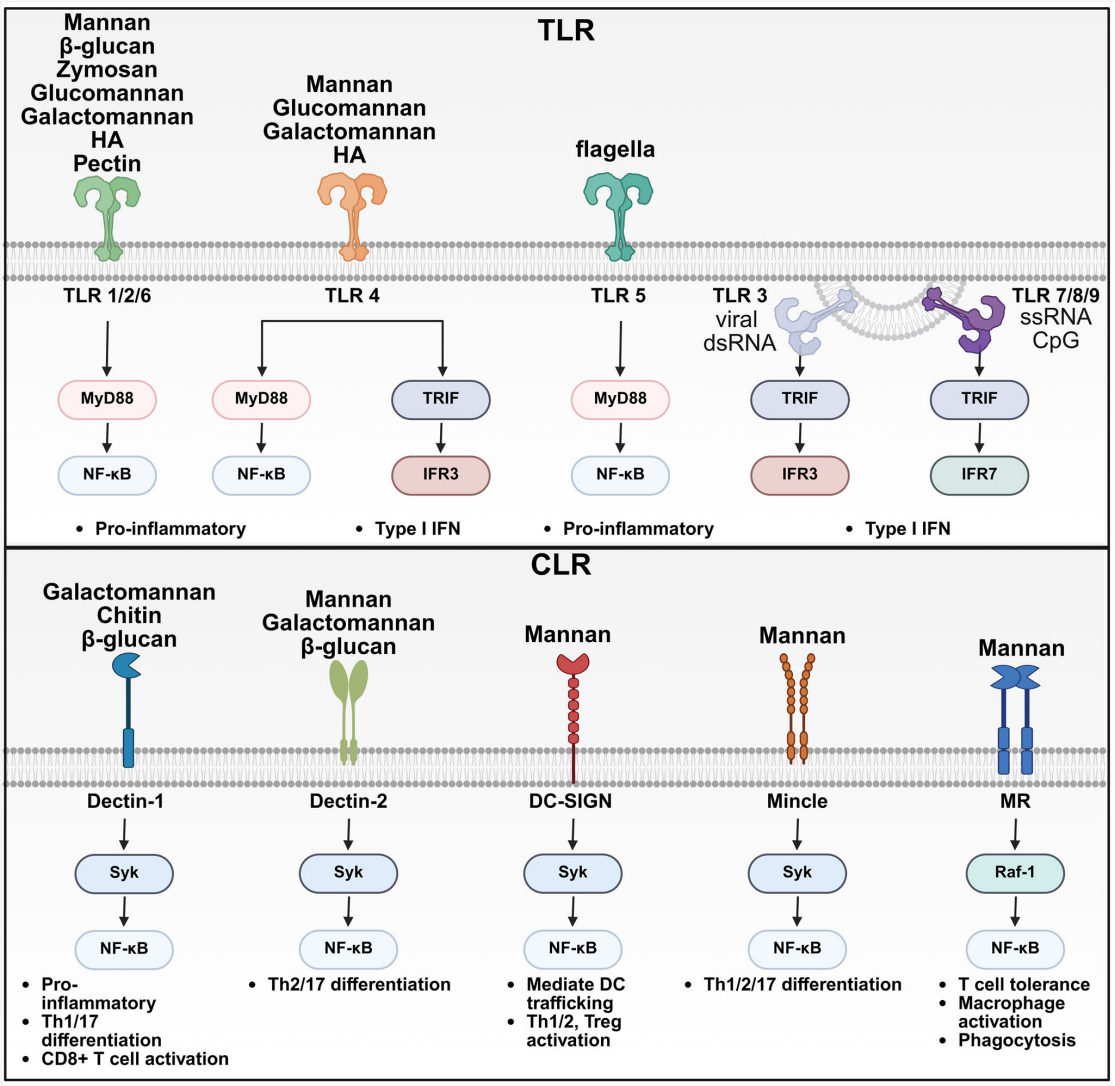
Pattern recognition receptors (PRRs) induce specific immune responses by recognition of polysaccharides as pathogen‐associated molecular patterns. Polysaccharides interact with PRRs such as Toll‐like receptors (TLRs) and C‐type lectin‐like receptors (CLRs). TLRs are broadly classified based on their cellular localization, with TLR1, TLR2, TLR4, TLR5, and TLR6 expressed on the cell surface, while TLR3, TLR7, TLR8, and TLR9 are primarily localized to intracellular membranes. CLRs, including Dectin‐1, Dectin‐2, DC‐SIGN, Mincle, and the mannose receptor (MR), represent another major class of PRRs. Interactions between polysaccharides and these PRRs can trigger the activation of immune‐related signaling cascades, leading to the transcription of genes regulated by pathways such as NF‐κB, IRF3, and IRF7.

In contrast to mice with twelve TLRs, ten different TLRs can be found in humans on the membrane of the cell surface, endosomes, or both. Among them, TLR‐2 and TLR‐4 are known for their ability to recognize polysaccharides. Notably, the polysaccharide‐based microbial components recognized by TLR2 include peptidoglycan, lipoarabinomannan, phospholipomannan, zymosan, and β‐Glycan derived from various origins, including gram‐positive and negative bacteria, mycobacteria, and fungi. This recognition ultimately results in the secretion of proinflammatory cytokines such as IL‐6, IL‐12p40, and TNF via NF‐kB activation mediated by MyD88‐dependent signaling pathways.^[^ ^45^, ^60^ ^]^ Another crucial receptor for polysaccharides is TLR‐4, which can be activated by most lipopolysaccharides (LPS) derived from Gram‐negative bacteria^[^ ^71^ ^]^ as well as fungal mannan and various other polysaccharides.^[^ ^60^ ^]^ Unlike TLR2 or other TLRs, TLR4 can promote secretion of type I IFNs and activation of NF‐kB through two signaling pathways: MyD88 and Toll/interleukin‐1 receptor (IL‐1R) domain‐containing adaptor inducing IFN‐β pathways (**Figure** **2**).^[^ ^72^ ^]^

##### CLRs

CLRs are another type of surface PRRs that can recognize specific immunostimulants with carbohydrate structures through carbohydrate recognition domains (CRDs) with Ca^2+^ dependence.^[^ ^73^ ^]^ They are classified into type I and type II transmembrane proteins, including Dectin‐1, Dectin‐2, macrophage‑inducible C‑type lectin (MINCLE), mannose receptor (MR, CD206), dendritic cell‐specific intercellular adhesion molecule‐3‐grabbing non‐integrin (DC‐SIGN), and so forth. CLRs are primarily located on cell membranes, functioning as antigen receptors.^[^ ^20^, ^74^, ^75^ ^]^ The signaling of CLR involved a noncanonical immunoreceptor tyrosine‐based activation motif (ITAM) recruiting spleen tyrosine kinase (Syk). This results in NF‐kB activation, which subsequently leads to T cell differentiation, phagocytosis, and proinflammatory cytokine release.^[^ ^20^, ^74^, ^76^ ^]^

Dectin‐1 is a type II transmembrane receptor, commonly recognized as β‐glucan receptor.^[^ ^77^ ^]^ Dectin‐1 can be found in cells of myeloid lineage, including macrophages/monocytes, neutrophils, and DCs, and cells of lymphoid lineage, such as γδ T cells.^[^ ^78^, ^79^, ^80^ ^]^ Dectin‐1, with its single CRD, selectively recognizes β‐ (1 → 3)/(1 → 6) – glucans present in the cell walls of specific pathogens such as fungi and bacteria and notably exhibits Ca^2+^‐independent ligand recognition due to the absence of a Ca^2+^‐binding site in its CRD.^[^ ^78^, ^80^, ^81^, ^82^ ^]^ Activation of SYK‐caspase recruitment domain‐containing protein 9 (CARD9) pathway subsequently leads to activation of NF‐kB. The downstream responses of this encompass phagocytosis,^[^ ^83^ ^]^ generation of reactive oxygen species (ROS),^[^ ^84^, ^85^ ^]^ and production of inflammatory cytokines and chemokines such as TNF‐α, C‐X‐C motif chemokine ligand (CXCL)2, IL‑1β, IL‐2, IL‑6, IL‐8, IL‑12, and IL‑23.^[^ ^74^, ^80^, ^84^, ^85^, ^86^, ^87^ ^]^ Beyond the influence on innate immunity, Dectin‐1 signaling coordinates adaptive immune system in terms of native CD4^+^ T cells differentiation into Th 1 or 17,^[^ ^83^ ^]^ and CD8^+^ T cells activation.^[^ ^80^, ^88^ ^]^

Dectin‐2, a type II transmembrane receptor, has a high specificity for recognition of mannose polysaccharides. Dectin‐2 is primarily located on tissue macrophages and inflammatory monocytes, however mouse Dectin‐2 can be found on Kupffer cells, Langerhans cells, and certain DCs as well.^[^ ^89^ ^]^ Despite the lack of intracellular signaling motifs, Dectin‐2 binds to the common Fc receptor γ subunit (FcRγ) containing an ITAM, thereby triggering Syk kinase indirectly and activating NF‐kB.^[^ ^90^ ^]^ This results in the production of cytokines and chemokines such as TNF‐α, IL‐1, CXCL‐1, or IL‐6.^[^ ^74^, ^91^ ^]^ Association of Dectin‐2 with Th2 immune responses and Th17 differentiation has also been studied.^[^ ^89^, ^92^, ^93^, ^94^, ^95^, ^96^, ^97^ ^]^

Mincle, Macrophage‑inducible C‑type lectin, is a type II transmembrane receptor known for its ability to recognize glycolipids,^[^ ^74^, ^98^ ^]^ mainly expressed on cells such as macrophages, monocytes, DCs, and PMN.^[^ ^98^, ^99^ ^]^ Mincle binds ligands, including both DAMPs and PAMPs, at CRD and cholesterol/protein interaction sites, initiating signaling through the ITAM‐containing FcRγ.^[^ ^98^, ^100^, ^101^ ^]^ The subsequent signaling pathways involve Syk, Card9‐B‐cell lymphoma/leukemia 10‐mucosa‐associated lymphoid tissue lymphoma translocation protein 1, and NF‐kB, resulting in production of inducible NO synthase and expression of cytokines and chemokines, including TNF, IL‐6, granulocyte colony‐stimulating factor, IL‐10, macrophage inflammatory protein‐2 or CXCL1 and CXCL2.^[^ ^74^, ^98^, ^101^, ^102^ ^]^ Initially believed to solely elicit proinflammatory response, Mincle, upon further investigation, was revealed to also promote the production of anti‐inflammatory cytokines while interfering with pro‐inflammatory cytokine production.^[^ ^98^, ^99^, ^103^, ^104^ ^]^ Mincle is also associated with Th1, Th2 and Th17 immune responses.^[^ ^98^ ^]^

MR is a type I integral membrane receptor expressed in macrophages, monocytes, brain astrocytes, microglial cells, some endothelial cells, and some DCs.^[^ ^105^, ^106^ ^]^ Based on the type of activated MR‐expressing cells, MR expression level is differentially regulated by LPS, transcription factor peroxisome proliferator‐activated receptor‐γ prostaglandins, cytokines such as IL‐10, IL‐4, IL‐13, and IFNγ.^[^ ^107^ ^]^ MR recognizes various glycans and microbial ligands with a particular high affinity for polysaccharides exhibiting numerous terminal d‐Man residues.^[^ ^105^, ^106^ ^]^ Two out of eight CRDs of MRs can recognize polysaccharides in a Ca^2+^‐dependent manner.^[^ ^105^, ^108^ ^]^ As of now, MR‐intrinsic signaling has yet to be reported due to its lack of known intracellular signaling motifs. Thus, the primary function attributed to MR is the internalization of extracellular materials for clearance and antigen presentation.^[^ ^107^ ^]^ MR is involved in induction of T cell tolerance, stimulation of inflammatory response in macrophages, and clearance of pathogens through phagocytosis.^[^ ^107^, ^109^ ^]^

DC‐SIGN, dendritic cell‐specific intercellular adhesion molecule (ICAM)3‐grabbing nonintegrin,^[^ ^110^ ^]^ is a type II membrane receptor which can be found on various types of DCs, including interstitial DC, monocyte, and CD34^+^ derived DC, and a small subset of CD14^+^ peripheral blood DC.^[^ ^111^, ^112^ ^]^ DC‐SIGN primarily recognizes high mannose N‐linked glycans located on various viruses, bacteria, parasites, and fungi through its C‐terminal CRD^[^ ^113^, ^114^ ^]^ in a calcium‐dependent manner. This pathogen recognition initiates signaling that modulates phagocytosis, suppresses TLR‐induced maturation of DCs, and facilitates pathogen internalization.^[^ ^112^ ^]^ DC‐SIGN also binds to ICAM3 and ICAM‐2, which promotes interaction between DC and naïve T lymphocytes for T cell proliferation and mediates DC trafficking.^[^ ^111^ ^]^ Additionally, DC‐SIGN interactions with mannose or fucose carbohydrates are involved in the modulation of Th1, Th2, and Treg cell responses (Figure 2).^[^ ^115^ ^]^

#### 3.2.2 Targeting Cytosolic PRR Pathways

Other PRR pathways involved in recognition of polysaccharides include cytosolic PRR pathways such as NLR and RLR. NOD‐like receptor pyrin domain‐containing protein 3 (NLRP3) is part of the NLR family that can form inflammasome,^[^ ^116^ ^]^ along with the adaptor molecule Apoptosis‐associated‐speck‐like protein containing a caspase recruitment domain and caspase‐1.^[^ ^117^ ^]^ NLR pathway has been identified to be activated by polysaccharides such as β glucans and chitosan, resulting in the initiation of antiviral and antimicrobial immune responses.^[^ ^116^, ^117^ ^]^ Activation of cytoplasmic NLRP3 inflammasome by β glucans is mediated by dectin‐1/Syk signaling pathway with the involvement of the lysosomal cathepsin B protease, ROS formation, and potassium efflux. NLRP3 activation ultimately leads IL‐1β secretion and proinflammatory responses in human macrophages through caspase‐1‐dependent cleavage of pro‐IL‐1β to an active IL‐1β.^[^ ^117^, ^118^ ^]^

RLRs, located in the cytosol, constitute a protein family comprising RIG‐I, melanoma differentiation‐associated protein 5 (MDA5), and laboratory of genetics and physiology 2. The primary role of RLR in recognition of immunostimulatory RNAs is supported by the cooperative interaction between the central helicase domain and carboxy‐terminal domain present in all RLRs.^[^ ^119^ ^]^ The association between RLR and polysaccharides was proposed in a study, wherein β‐glucans were identified to upregulate the expression of RIG‐I and MDA5.^[^ ^120^ ^]^ Activation of CARDs located on RIG‐I and MDA5 subsequently mediates downstream signal transduction.^[^ ^119^ ^]^ By activating transcriptional factors such as IRF, NF‐kB, and activator protein 1, RIG‐I promotes secretion of IFN, inflammatory cytokines, and chemokines.^[^ ^121^, ^122^ ^]^

#### 3.2.3 Targeting Secreted PRRs

Secreted PRRs also play a crucial role in PAMP recognition and initiation of host immune responses. Secreted PRRs are released into the plasma after being produced by hepatocytes.^[^ ^123^ ^]^ Notably, mannan‐binding‐lectin (MBL), part of the collagenous lectin protein family,^[^ ^124^ ^]^ can recognize microbial cells. They then mark the microbes for either phagocytosis or elimination by the complement system.^[^ ^125^ ^]^ MBL, with the assistance of MBL‐associated serine proteases triggers the lectin activation pathway of complement.^[^ ^124^ ^]^ MBL also has the potential to engage with cell surface receptors to facilitate opsonophagocytosis through a pathway independent of complement. Functional MBL binds to the pathogens based on the steric specificity of MBL and the spatial arrangement of carbohydrate ligands on the pathogen's surface.^[^ ^126^ ^]^ MBL is associated with the modulation of inflammation through the production of cytokines such as TNF‐α, IL1β, and IL‐6 by monocytes.^[^ ^127^ ^]^

### 3.3 Improving Immunogenicity of Polysaccharides

The immunological behavior of polysaccharides is influenced by various factors, including their biological origin, structural features, monosaccharide composition, molecular conformation, size, and functional moieties. However, their broader application in immunotherapy has been limited, primarily due to their inherently low immunogenicity.

The molecular weight (MW) of polysaccharides, typically spanning from several tens to thousands of kilodaltons, is a critical parameter influencing their interactions with biological components, including receptors, immune cells, and various organs.^[^ ^128^ ^]^ Polysaccharides with higher molecular weights are generally linked to enhanced immune responses,^[^ ^129^ ^]^ as their size influences key biological processes such as cellular uptake, retention time, degradation, and adsorption kinetics, especially within the gastrointestinal (GI) environment.^[^ ^130^ ^]^ Their extended chain length and flexible conformations enable multivalent presentation of carbohydrate motifs, which markedly enhances the probability of simultaneous PRR engagement compared with small‐molecule or short‐chain agonists. For example, high‐MW β‐glucans (>500 kDa) can crosslink Dectin‐1 on macrophages and dendritic cells, amplifying Syk–CARD9–NF‐κB signaling, upregulating co‐stimulatory molecules (CD80/CD86), and promoting IL‐23‐driven innate memory responses,^[^ ^131^ ^]^ whereas lower‐MW fragments (<10 kDa) remain immunologically inactive.^[^ ^132^ ^]^ This multivalency‐driven receptor clustering underlies one of the major advantages of high‐MW polysaccharides in spatiotemporal immune regulation. When multiple receptor binding sites are simultaneously occupied by a single polysaccharide chain or nanoparticle, receptor crosslinking occurs on the cell surface, leading to enhanced receptor internalization and PRR‐mediated endocytosis. Such receptor clustering is a critical trigger for downstream signaling cascades, including activation of the NF‐κB and mitogen‐activated protein kinase (MAPK) pathways, which promote the transcription of cytokines and co‐stimulatory molecules.^[^ ^33^ ^]^ For instance, mannan‐coated nanocapsules have been reported to activate multiple immune receptors, including Dectin‐2 and TLR‐4. These multivalent PRR engagement promote scavenger receptor– and clathrin‐mediated uptake by dendritic cells, leading to receptor clustering, enhanced downstream signaling, and subsequent activation of Th17‐polarizing immune responses.^[^ ^133^ ^]^ These examples highlight how MW‐dependent chain length and substituent chemistry cooperatively tune receptor multivalency, endocytosis efficiency, and signal amplitude.

**Figure 3:**
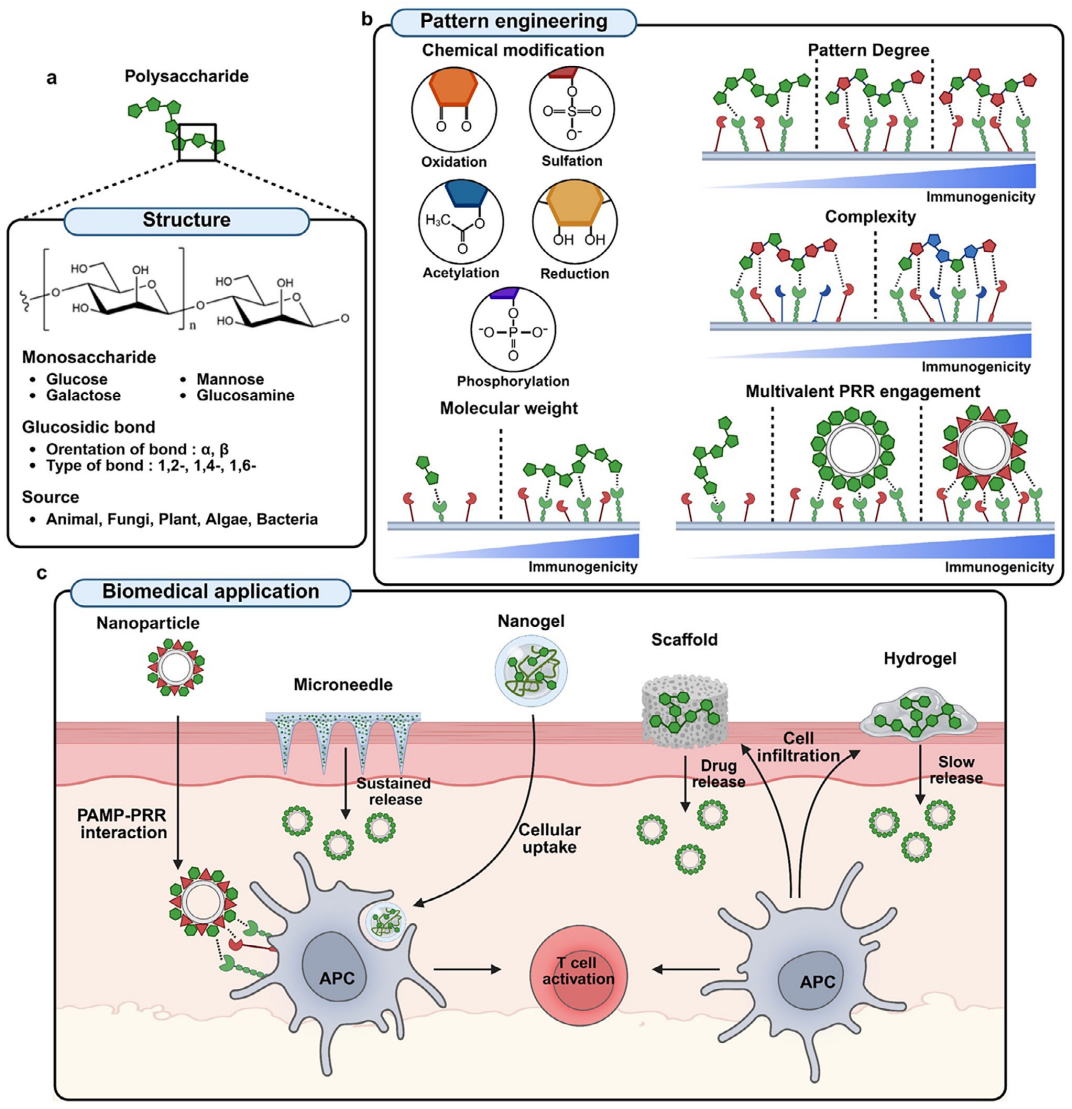
A) Pattern structure of polysaccharide. Polysaccharides consist of monosaccharides such as glucose and galactose connected by glycosidic bonds with specific orientation, bond types, and sources. B) Pattern engineering. Molecular weight serves as a key physicochemical parameter influencing pattern recognition receptor (PRR) engagement, with higher molecular weights generally associated with enhanced immunogenicity. Chemical modification of polysaccharides to introduce defined structural patterns can further modulate PRR interactions, depending on the degree and complexity of the patterning. Additionally, particulation enables multivalent engagement of PRRs, thereby amplifying immunostimulatory effects. C) Biomedical application. Polysaccharide‐based platforms for biomedical applications include nanoparticles, nanogels, microneedles, scaffolds, and hydrogels. Nanoparticles can activate immune responses by engaging APCs through PAMP–PRR interactions. Microneedles are designed to encapsulate therapeutic agents, enabling sustained release over time. Nanogels are efficiently taken up by APCs, facilitating intracellular delivery. Scaffolds and hydrogels can be loaded with therapeutic agents within their porous structures, allowing for controlled release or interaction with infiltrating immune cells. Upon activation, APCs initiate T cell responses, thereby contributing to the modulation and enhancement of the immune system.

Chemical modification serves as a rational strategy to expand the structural and functional diversity of polysaccharides, enabling precise control over their physicochemical behavior and biological recognition. The introduction of distinct functional groups, such as carboxymethylation,^[^ ^134^ ^]^ sulfation,^[^ ^135^ ^]^ phosphorylation, and acetylation,^[^ ^136^, ^137^ ^]^ reshapes polymer conformation, charge distribution, and hydration characteristics thereby modulating how polysaccharides interact with PRRs on immune cells. These modifications affect the strength of ligand–receptor interaction (affinity).^[^ ^33^ ^]^ For instance, negatively charged or hydrophilic substitutions improve aqueous solubility and expand molecular flexibility, facilitating receptor accessibility and hydrogen‐bonding interactions with CRD.^[^ ^33^, ^138^ ^]^ In contrast, hydrophobic or bulky groups promote chain self‐association or folding, which can enhance localized receptor clustering but may also reduce ligand exposure depending on degree of substitution (DS).^[^ ^135^ ^]^ Furthermore, the rational use of chemical modification allows the tuning of spatial organization, both of which are crucial for selective and efficient PRR engagement.^[^ ^33^ ^]^ By modulating chain stiffness or DS, polysaccharides can be engineered to optimize PRR clustering and affinity, thereby modulating immune activation while preventing overstimulation.^[^ ^139^ ^]^ For instance, acetylated glucomannan with 1.8 DS (acGM‐1.8) exhibits increased hydrophobicity and partial inter‐chain rearrangement due to acetyl substitution, which promotes degree‐dependent self‐assembly into nanoscale particles. This conformational transition improves the spatial presentation of carbohydrate motifs and enhances TLR2‐mediated signaling, leading to elevated pro‐inflammatory cytokine release and promoting polarization of macrophage toward an antitumor phenotype.^[^ ^136^ ^]^ Similarly, inulin acetylation converts the polymer from a soluble chain into a self‐assembling nanoparticle, producing a multivalent carbohydrate surface that strengthens interactions with the TLR4/myeloid differentiation factor 2(MD‐2) complex. The resulting particles behave as semi‐synthetic TLR4 agonists, eliciting PRR clustering and coordinated chemokine and cytokine production.^[^ ^137^ ^]^ Benzoylation further refines TLR4 targeting by introducing aromatic acyl groups that interact with hydrophobic residues in the MD‐2 binding pocket through π–π stacking, hydrogen bonding, and hydrophobic contacts, thereby stabilizing receptor dimerization and facilitating downstream dendritic‐cell signaling.^[^ ^140^ ^]^ Conversely, sulfation can bias polysaccharide activity toward immune modulation. Glycopolymers containing 3‐O‐sulfated galactose (SO_4_‐3‐Gal) preferentially interact with the cysteine‐rich domain of CD206, forming stable endosomal complexes that inhibit CD206 recycling and attenuate CD206‐mediated immune activation (Figure 3b).^[^ ^141^ ^]^

Beyond receptor engagement, polysaccharides possess inherent environmental responsiveness, allowing their physicochemical properties and assembly behavior to adapt dynamically to the biological microenvironment. Such responsiveness allows microenvironment‐dependent release of immunologically active cargo, aligning the timing and location of immune stimulation with the physiological property. Polysaccharides with ionizable groups (e.g., carboxyl, amino, or sulfate) exhibit pH‐dependent protonation–deprotonation, allowing site‐specific swelling, collapse, or disassembly under acidic or neutral conditions characteristic of endosomes, inflamed tissue, or the GI tract.^[^ ^142^ ^]^ For instance, chitosan nanoparticles remain stable at neutral pH but dissolve in acidic environments due to protonation of amino groups, facilitating targeted antigen or drug release in endosomes or the stomach.^[^ ^143^ ^]^

Polysaccharides can also undergo enzyme‐mediated degradation, allowing spatiotemporally restricted immune activation at sites rich in specific enzymes. For example, dextran and mannan are cleaved by glycosidases abundant in lysosomes and intestinal microbiota, leading to intracellular or colon‐targeted delivery of immunomodulatory molecules.^[^ ^144^, ^145^ ^]^ Likewise, alginate–chitosan hybrid hydrogels exhibit reversible swelling and degradation triggered by both pH and enzymatic activity, ensuring sustained antigen exposure to DCs. This continuous exposure prolongs DC maturation and cytokine secretion, reinforcing the immunostimulatory microenvironment and enhancing the adaptive immune response.^[^ ^146^ ^]^

Building upon enzymatic responsiveness, polysaccharides also display redox sensitivity that allows further refinement of microenvironment‐responsive immune control. Inflamed and tumor tissues often possess elevated levels of ROS and reducing agents such as glutathione. Polysaccharides functionalized with redox‐labile linkages, including disulfide, thioether, and boronic ester bonds, undergo selective cleavage in these environments, providing precise control over antigen or drug release.^[^ ^147^, ^148^ ^]^ For example, boronic ester‐modified polysaccharides are cleaved by ROS, leading to hydrophilic conversion and rapid payload liberation,^[^ ^149^ ^]^ while thiolated pullulan or dextran derivatives remain stable in systemic circulation but disassemble under intracellular reducing conditions. The resulting on‐site release within antigen‐presenting cells enhances endosomal escape and cross‐presentation, leading to efficient cytotoxic T‐cell activation and precisely localized immune stimulation.^[^ ^150^ ^]^ Collectively, the enzyme‐ and redox‐responsive adaptability of polysaccharides integrates seamlessly with their MW‐driven multivalency, forming a unified framework of spatiotemporal immune modulation. These combined physicochemical features enable site‐specific activation, prolonged immune signaling, and controlled antigen presentation, establishing polysaccharides as versatile platforms for next‐generation vaccine and immunotherapy design.

To improve the immunostimulatory capacity of polysaccharides and optimize drug delivery efficiency, a range of biomedical platforms such as nanoparticle, hydrogel, and scaffold has been engineered, utilizing the inherent bioactivity and adaptable structural properties of polysaccharide. Nanogels are nanosized hydrogel systems formed from crosslinked, hydrophilic polymer networks with a high water‐retention capacity, enabling the encapsulation of small bioactive compounds. Owing to their favorable size and surface characteristics, they are readily internalized by antigen‐presenting cells, supporting their utility as efficient delivery vehicles in immunotherapy (Figure 3c).

Polysaccharides, owing to their high MW, biodegradability, and structural versatility, are uniquely positioned to function as dual‐purpose platforms serving both as antigen carriers and self‐adjuvanting agents in vaccine formulations.^[^ ^33^ ^]^ Their extended chain architecture provides ample surface area and functional sites for antigen conjugation or encapsulation, while the abundant hydroxyl, carboxyl, or amino groups enable chemical modification for fine‐tuning charge, solubility, and crosslinking density.^[^ ^151^ ^]^ These physicochemical attributes facilitate the assembly of polysaccharides into nanostructured carriers (e.g., nanogels, microparticles, or nanocapsules) capable of protecting antigens from enzymatic degradation and ensuring controlled release at mucosal or intracellular sites. Such macromolecular assemblies extend residence time at epithelial interfaces and preserve antigen integrity, features essential for achieving robust oral and mucosal vaccine delivery.

Beyond serving as passive carriers, polysaccharides exhibit intrinsic adjuvanticity by coordinating their molecular architecture with the structural recognition patterns of PRRs such as Dectin‐1, CD206, and TLR2/4. Their repetitive saccharide motifs and spatially organized glycosidic linkages mirror the molecular topologies of natural PAMPs, enabling multivalent and geometrically compatible interactions with receptor‐binding pockets. This structural congruence facilitates receptor clustering and crosslinking on immune cell membranes, which enhances endocytosis, prolongs PRR signaling, and drives cytokine and co‐stimulatory molecule expression.

By aligning the molecular conformation of natural polysaccharide scaffolds with PRR‐binding geometries, these materials achieve precise control over signal intensity and duration, translating structural recognition into functional immune modulation. Functionally, this coordination enhances dendritic‐cell maturation and cytokine polarization, thereby reducing the antigen dose required for effective priming. Importantly, because the same polysaccharide matrix that carries the antigen also interfaces with PRRs, such systems act as self‐adjuvanting nanocarriers that synchronize antigen delivery and innate immune activation within shared endosomal compartments. This spatiotemporal orchestration aligns MHC‐II antigen processing and, when engineered for endosomal escape, promotes MHC‐I cross‐presentation, resulting in robust and balanced T‐cell activation with minimized systemic inflammation.

### 3.4 Trained Immunity

#### 3.4.1 Mechanism of Trained Immunity

Conventionally, the concept of immune memory was thought to be exclusive to the adaptive immune system. However, studies on the BCG vaccine, originally developed for tuberculosis, have revealed that vaccines can also enhance protection against unrelated infections.^[^ ^152^, ^153^ ^]^ The implications of these discoveries led to the concept of trained immunity, introduced in 2011,^[^ ^154^, ^155^ ^]^ which proposes that innate immune cells can retain immunologic memory.^[^ ^156^ ^]^ This enhanced response is driven by metabolic reprogramming and epigenetic changes in myeloid cells, enabling a more potent immune response to subsequent stimuli.^[^ ^154^, ^157^, ^158^, ^159^, ^160^, ^161^, ^162^ ^]^

**Figure 4:**
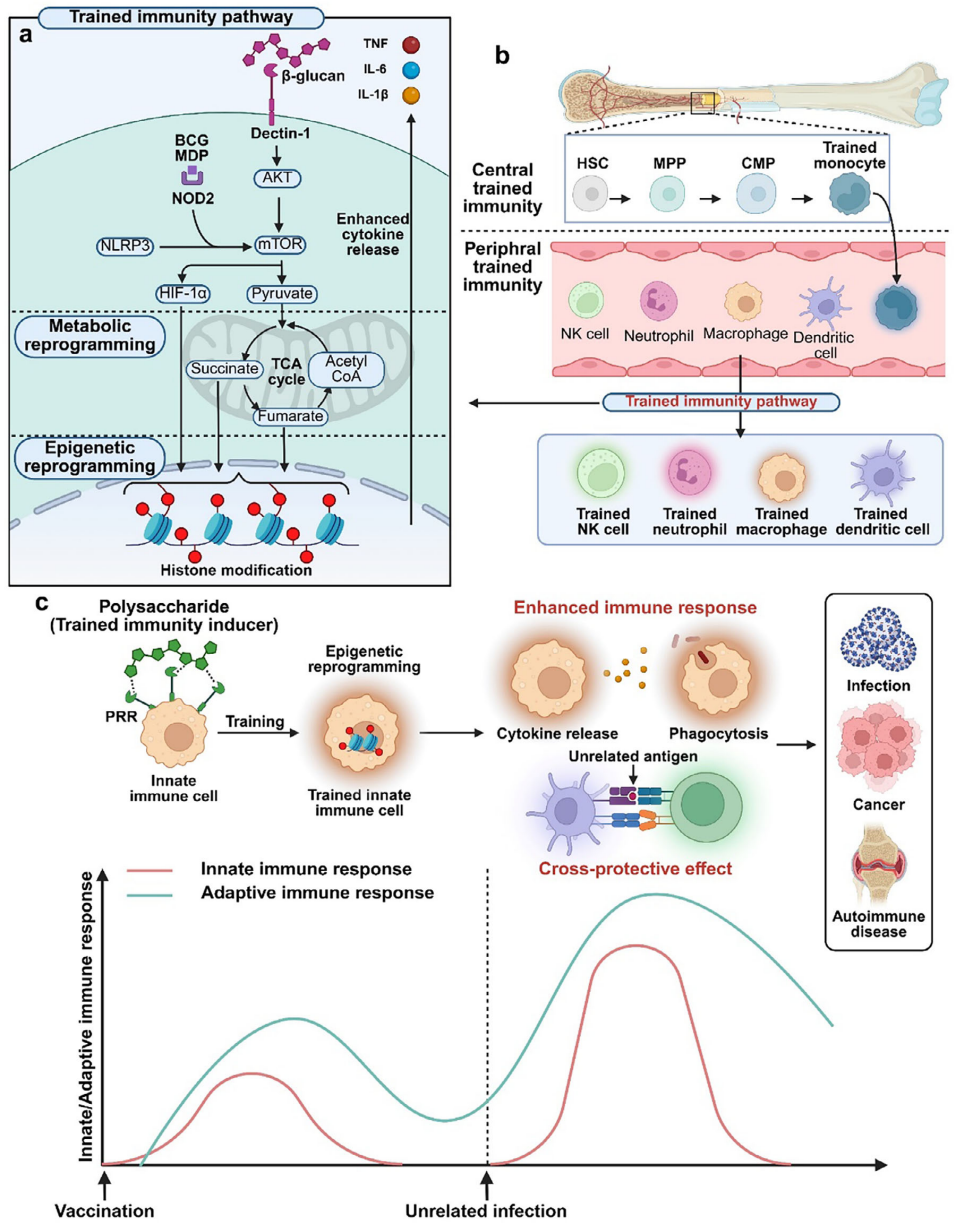
Mechanism of trained immunity and trained immunity adjuvanted vaccine effect. A) Trained immunity is the process, which innate cells acquire immune memory through trained immunity pathway. BCG, MDP, and β‐glucan interact with their specific receptors, triggering an amplified secretion of IL‐6, TNF‐α, and IL‐1β via a sequential cascade involving metabolic and epigenetic reprogramming. B) The classification of central trained immunity and peripheral trained immunity is determined by origination from either myeloid precursor cells in bone marrow or circulating innate immune cells in the bloodstream, respectively. C) Trained immunity adjuvanted vaccines prompt both cellular and humoral immunity by eliciting vaccine antigen‐specific T cell response and instigating trained state within innate immune cells. Trained innate immune cells foster enhanced antigen‐specific adaptive immunity against subsequent related pathogen infection and counters by cross‐protection against unrelated pathogen infections.

Trained immunity, initiated in innate immune cells upon encountering pathogens, is driven by the recognition of PAMPs such as β‐glucan and BCG by PRRs including dectin‐1 and NOD2. This interaction induces chromatin remodeling in monocytes and macrophages, thereby establishing a heightened state of immune responsiveness.^[^ ^157^, ^163^, ^164^, ^165^ ^]^ During the initiation of trained immunity, the protein kinase B (AKT)/mammalian target of rapamycin (mTOR) signaling axis triggers a metabolic shift, wherein cellular energy production transitions from oxidative phosphorylation to aerobic glycolysis.^[^ ^166^ ^]^ This leads to accumulation of key tricarboxylic acid cycle metabolites, including fumarate, succinate, mevalonate, and α‐ketoglutarate which play a crucial role in trained immunity by epigenetic reprogramming, including the regulation of histone‐modifying enzymes and gene expression.^[^ ^167^ ^]^ For instance, succinate and fumarate contribute to inhibiting histone demethylases, leading to increased histone methylation, while α‐ketoglutarate supports an anti‐inflammatory macrophage phenotype histone H3 lysine 27 demethylase‐mediated epigenetic reprogramming.^[^ ^168^ ^]^ These epigenetic alterations modulate chromatin structure to facilitate the accessibility of gene promoter regions for enhancing secretion of inflammatory cytokines such as IL‐6, IL‐1β, and TNF, thereby bolstering the immune response against unrelated pathogens, PAMP stimuli,^[^ ^163^ ^]^ as well as parasitic^[^ ^169^ ^]^ and viral pathogens (**Figure** **4**a).^[^ ^170^ ^]^

Trained immunity is generally categorized into central and peripheral types, depending on the location and underlying mechanisms. Central trained immunity describes the sustained functional reprogramming of hematopoietic stem and progenitor cells within the bone marrow. Recent research has revealed that β‐glucan and BCG can epigenetically reprogram bone marrow progenitor cells, enhancing myelopoiesis through IFN‐γ‐dependent pathways^[^ ^171^ ^]^ Moreover, β‐glucan has been found to enhance myelopoiesis by enhancing the proliferation of myeloid‐biased hematopoietic stem cells and myeloid‐biased pluripotent progenitor cell 3 through induced metabolic changes such as increased glycolysis and mitochondrial activity.^[^ ^172^ ^]^ In contrast, peripheral trained immunity arises in differentiated innate immune cells, including monocytes, macrophages, and NK cells, residing in peripheral tissues. Exposure to specific PAMPs or DAMPs induces epigenetic and metabolic reprogramming in these mature cells, thereby enhancing their functional responses, including elevated cytokine production and more effective pathogen clearance during subsequent encounters.^[^ ^165^ ^]^ Unlike central trained immunity, peripheral trained immunity is typically more short‐lived and confined to local tissues, yet it can substantially enhance the initial immune response to infections or tissue injury (Figure 4b).^[^ ^173^ ^]^

Polysaccharides, including β‐glucan, mannan, chitin, and arabinoxylans, are known to act as PAMPs and have been identified as inducers of trained immunity. β‐glucan recognized by Dectin‐1 triggers a signaling cascade involving AKT, mTOR, and hypoxia‐inducible factor 1‐alpha, leading to metabolic reprogramming for trained immunity.^[^ ^166^ ^]^ This improves the phagocytosis of cancer cells by macrophages, stimulates the recruitment and maturation of dendritic cells, and promotes T cell activation. Furthermore, it boosts the circulation of trained monocytes/macrophages, facilitating their differentiation into M1‐like macrophages within tumor microenvironments.^[^ ^174^, ^175^ ^]^ For mannan, the allergoid‐mannan conjugates utilize mannan to specifically target DCs by binding to the mannose receptor and DC‐SIGN. This interaction promotes allergen uptake and enhances the expression of IL‐10 and programmed death‐ligand 1 (PD‐L1). Additionally, the conjugates reprogram monocyte differentiation, inducing the tolerogenic DCs through epigenetic and metabolic reprogramming to facilitate the generation of allergen‐specific FOXP3^+^ Tregs.^[^ ^176^ ^]^ Chitin triggers trained immunity in monocytes, promoting increased production of cytokines such as TNF‐α and IL‐6 upon secondary stimulation with TLR ligands and commensal bacteria and fungi. This chitin‐induced trained immunity is closely linked to its internalization and subsequent phagosome acidification, which is crucial for enhanced antimicrobial activity in the host.^[^ ^177^ ^]^ Arabinoxylans sourced from rice and wheat were shown to stimulate trained immunity in human macrophages by activating PRRs, particularly Dectin‐1 and complement receptor 3 (CR3). The degree of immune training and resilience induced was found to be dependent on the fiber size and solubility of arabinoxylans.^[^ ^178^ ^]^ Polysaccharides induce trained immunity by engaging specific PRRs, making them promising candidates as adjuvants for trained immunity vaccines.

#### 3.4.2 Therapeutic Approach of Trained Immunity‐Inducing Vaccine

Since Buffen and colleagues uncovered the mechanisms of trained immunity triggered by BCG in bladder cancer treatment,^[^ ^158^ ^]^ this phenomenon has also been observed in measles,^[^ ^159^ ^]^ influenza,^[^ ^160^, ^161^ ^]^ and even the latest mRNA vaccine against Covid‐19.^[^ ^162^ ^]^ This indicates that vaccines developed for specific diseases could potentially be repurposed to treat other diseases by leveraging the properties of trained immunity.

Trained immunity‐based vaccines (TIbV) have emerged as promising tools that provide not only pathogen‐specific protection but also defense against antigenically unrelated secondary infections. This broad‐spectrum immune enhancement, referred to as heterologous protection, is attributed to the ability of TIbV to epigenetically and metabolically reprogram innate immune cells such as monocytes, macrophages, and NK cells.^[^ ^156^ ^]^ Agents like β‐glucan, BCG, and synthetic analogs initiate such reprogramming, leading to a sustained, heightened immunological state marked by increased production of pro‐inflammatory cytokines,^[^ ^179^ ^]^ enhanced generation of ROS,^[^ ^180^ ^]^ and improved recognition of invading pathogens,^[^ ^181^ ^]^ independent of classical immune memory.

When challenged with a heterologous pathogen, these trained innate cells elicit rapid and potent responses that prevent early pathogen expansion. Additionally, APCs conditioned by TIbV demonstrate augmented capabilities in antigen uptake, processing, and presentation, irrespective of antigen specificity. This enhancement facilitates more efficient activation of T and B cells upon secondary infection, thereby accelerating the initiation of adaptive immunity even in the absence of prior exposure to the new pathogen.^[^ ^182^ ^]^ Furthermore, TIbV‐induced inflammatory environments support the expansion and functional activation of bystander memory T cells, those that are not specific to the primary antigen but can be stimulated through cytokine signaling during secondary challenges.^[^ ^183^ ^]^ The long‐term effects of trained immunity may also stem from reprogramming at the level of hematopoietic stem and progenitor cells, resulting in a persistent bias toward myelopoiesis and a pro‐inflammatory phenotype.^[^ ^172^ ^]^ This systemic reconfiguration underlies the capacity of TIbVs to maintain an immune‐ready state with cross‐protective potential against diverse microbial threats (Figure 4c).^[^ ^182^ ^]^

Leveraging trained immunity in vaccines offers a strategy to mitigate the impact of pandemics like Covid‐19 by capitalizing on the non‐specific protective effects of trained immune vaccines during the development gap.^[^ ^182^ ^]^ Additionally, when used as an adjuvant, trained immunity exhibits traits of both innate and adaptive immune memory, providing robust protection against specific antigens via adaptive immunity and cross‐protection against non‐specific antigens through trained immunity.

## 4 Biological Barriers for Vaccine Development

**Figure 5:**
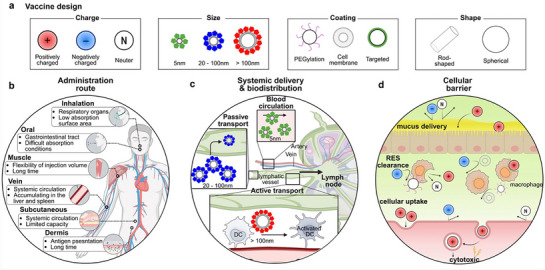
Integrated analysis of nanoparticle properties impacting administration routes and draining lymph node dynamics. A) Trends according to nanoparticle properties In systemic circulation, rod‐shaped nanoparticles are more readily excreted than spherical nanoparticles, and positively charged or uncoated nanoparticles are easily cleared by macrophages. Rod‐shaped or neutral nanoparticles can easily penetrate tumors, and positively charged or coated nanoparticles can easily pass through mucosal barriers. B) Overview of administration route. Due to different characteristics depending on the administration route, the therapeutic effect varies depending on the administration route. C) Draining lymph node and blood. Afferent lymphatic vessels are connected to the subcapsular sinus, where most DCs reside. Through afferent lymphatic vessels, NPs can move to lymph nodes through passive transport (∼20‐100 nm) or cell‐mediated transport. Activated DCs by NPs prime T cells for adaptive immune responses through signals 1, 2, and 3. D) Cellular barrier Negatively charged mucosal and cellular membranes repel negatively charged particles, while positively charged particles can adhere via electrostatic attraction, enhancing mucosal penetration and cellular uptake. However, excessive positive charge may cause membrane damage and cytotoxicity. PEGylation can help evade RES clearance, improving circulation time.

The efficacy of drugs greatly hinges on their ability to reach their intended site of action. Medications exhibiting high efficacy in laboratory settings may fail to produce desired effects within the body, often due to biological barriers that regulate access to tissues and organs. Among these barriers, the vascular system stands out as a prominent obstacle for pharmaceutical agents, as they typically rely on diffusion through blood vessels to reach their designated sites of action.^[^ ^184^ ^]^ The endothelial cells that line blood vessels act as a barrier controlling the movement of substances in and out of organs. The endothelium varies greatly among different organs, reflecting their unique physiological roles.^[^ ^185^ ^]^ Vaccines can be strategically designed to overcome challenges associated with various administration routes, systemic delivery, biodistribution, and cellular barriers by tuning physicochemical properties such as charge, size, surface coating, and shape (**Figure** **5**a). Tailoring these parameters enables optimization of vaccine performance and enhances immune targeting efficiency across different biological contexts.

### 4.1 Administration Route Barriers

Drugs can be delivered through multiple administration routes, each presenting distinct physiological environments and transport challenges. These routes, including oral, intravenous, subcutaneous, and intramuscular.present distinct barriers that affect how efficiently a therapeutic agent reaches its target site. Intravenous administration has the characteristics of direct delivery to systemic circulation without delay, and high drug bioavailability.^[^ ^186^, ^187^ ^]^ Due to these characteristics, APCs in the spleen robustly prime CD8^+^ T cells, enhancing the efficacy of cancer vaccines.^[^ ^186^ ^]^ When drugs circulate systemically, they are rapidly cleared by immune cells, leading to difficulties in prolonged accumulation at inflammatory sites and primarily accumulating in the liver and spleen, making delivery to desired tissues challenging.^[^ ^187^, ^188^, ^189^ ^]^ Intramuscular administration is often preferred due to its flexibility in injection volume and its ability to induce adaptive immunity through innate immune responses even with small amounts of antigens.^[^ ^189^, ^190^ ^]^ However, it takes time for the therapeutic effect to occur as the drug needs to be absorbed before entering the bloodstream.^[^ ^186^ ^]^ Subcutaneous administration penetrates through the extracellular matrix (ECM) and circulates throughout the body by capillaries or is transported to the lymphatic system.^[^ ^191^ ^]^ Due to its capability for self‐administration, it is known to be easily accessible and to induce strong cytotoxic T cell immunity in a melanoma model in mice.^[^ ^189^, ^191^ ^]^ Due to the restricted injection volume of 1.5–2 mL, it is challenging to inject therapeutically meaningful concentrations.^[^ ^191^ ^]^ Intradermal administration is known for its ability to utilize DCs and Langerhans cells present in the dermis and epidermis, which excel in presenting antigens through vaccines.^[^ ^189^ ^]^ However, due to the slow absorption and the requirement for the drug to reach the dermis for systemic circulation, it is primarily utilized in the form of local therapy for dermatological conditions.^[^ ^191^ ^]^ The oral administration route represents a highly desirable platform for vaccination due to its ease of use and potential to elicit mucosal and systemic immunity. However, effective antigen delivery through the GI tract remains challenging because of acidic pH, proteolytic enzymes, mucus entrapment, and tight epithelial junctions, all of which reduce antigen stability and mucosal uptake.^[^ ^192^ ^]^ Polysaccharide‐based adjuvants provide a rational strategy to overcome these barriers by integrating biophysical protection, mucosal transport, and innate immune activation within a single biomaterial framework.

Polysaccharides possess distinctive advantages for oral delivery by enabling therapeutic agents to overcome the complex physiological barriers of the GI tract. Their natural tolerance to acidic environments and enzymatic activity in the stomach allows for improved molecular stability and enhanced bioavailability compared with protein‐ or peptide‐based systems.^[^ ^193^ ^]^ Hydrogels and coating materials derived from polysaccharides such as alginate,^[^ ^194^, ^195^, ^196^ ^]^ chitosan,^[^ ^195^, ^197^ ^]^ inulin,^[^ ^198^, ^199^ ^]^ and xylan^[^ ^200^ ^]^ have been shown to maintain their structural integrity under gastric conditions and in the presence of digestive enzymes, thereby providing effective protection for encapsulated antigen. Polysaccharide assemble into mucoadhesive, pH‐responsive carriers that shield antigens and prolong residence at the intestinal surface, thereby enhancing the probability of uptake at mucosal immune sites.^[^ ^201^ ^]^ Chitosan nanoparticles transiently loosen epithelial tight junctions by modulating zonula occludens‐1 and occludin, thereby facilitating paracellular transport and improving antigen accessibility to immune‐inductive sites. Such physicochemical adaptability enables antigens to reach Peyer's patches or microfold cell (M cells), where immune priming is initiated.

Polysaccharides such as β‐glucan, mannan, and chitosan interact with pattern PRRs, including Dectin‐1, CD206, and TLR2/4 expressed on M cells and mucosal APCs. These multivalent carbohydrate–PRR interactions mediate receptor‐dependent endocytosis and transcytosis across M cells, enabling efficient delivery of antigen–polysaccharide complexes to the immune inductive microenvironment of Peyer's patches.^[^ ^202^, ^203^ ^]^ Among them, β‐glucan microparticles have been shown to bind Dectin‐1 on M‐cell surfaces, promoting selective uptake into intestinal lymphoid tissues and enhancing local immunoglobulin (Ig) A and systemic IgG responses.^[^ ^202^ ^]^ Also, calcium phosphate nanoparticles coated with polysaccharides such as alginate and chitosan and loaded with antigen significantly enhanced both mucosal (secretory immunoglobulin A, sIgA) and systemic (serum IgG) immune responses, accompanied by activation of intestinal PRRs, including TLR4 and Dectin‐1.^[^ ^204^ ^]^ Collectively, these studies demonstrate that polysaccharide–PRR coordination allows antigens and immune signals to co‐localize within the same endocytic pathway, achieving efficient mucosal immune priming and significantly improved oral vaccine efficacy.^[^ ^201^ ^]^

Inhalation and intranasal administration can target respiratory organs such as the lungs, where continuous drug absorption can be expected.^[^ ^187^ ^]^ Since the nasal cavity is known to be rich in DCs, it may induce potent local and systemic immune responses against infections.^[^ ^186^ ^]^ This allows drugs to invade the mucosa, effectively eliciting specific immune responses such as IgA production. The released IgA represents both systemic and mucosal immunity in the respiratory tract.^[^ ^186^ ^]^ Furthermore, it is known to induce the generation of CD4^+^ and CD8^+^ effector memory T cells as well as eliciting tissue‐resident memory T cell responses within the respiratory tract tissues.^[^ ^190^ ^]^ However, due to the relatively low absorption surface area and the potential for systemic distribution leading to accumulation in the central nervous system, these routes may carry a risk of inducing unintended effects (Figure 5b).^[^ ^187^, ^191^ ^]^

### 4.2 Systemic Delivery and Biodistribution

Systemic administration enables therapeutic agents to circulate throughout the body, but their distribution is influenced by physiological barriers and the physicochemical properties of the drug. Optimizing these properties is critical to enhance tissue targeting and therapeutic efficacy. Various features of the tumor microenvironment, including abnormal vasculature, interstitial fluid pressure, and density of the ECM, collectively contribute to the restricted permeation and penetration of nanoparticles (NPs).^[^ ^205^, ^206^, ^207^, ^208^ ^]^ Some of these NPs with features such as hydrodynamic diameters above 100 nm, rod‐shaped, near‐neutral charges, or inorganic material compositions are considered favorable for tumor accumulation.^[^ ^209^ ^]^ These properties help them take advantage of enhanced permeability and retention effect, where leaky tumor vasculature allows prolonged circulation and efficient NP extravasation into tumor tissues.^[^ ^210^, ^211^ ^]^

Lymph nodes house a significant population of immature APCs capable of initiating an immune response independently of migratory populations from peripheral tissues.^[^ ^212^ ^]^ Directing vaccine components to these lymph node‐resident APCs (passive transport), without the prolonged migration of APCs from the injection site to the lymph nodes (typically spanning 24–48 h), can expedite the activation of T cells. By meticulously adjusting the size,^[^ ^213^, ^214^ ^]^ shape,^[^ ^215^, ^216^ ^]^ charge, and hydrophobicity,^[^ ^217^, ^218^, ^219^ ^]^ efficient targeting of lymph nodes can be achieved without specific cell‐targeting ligands. NPs smaller than 5 nm swiftly enter the bloodstream, circulating systemically, while those sized between 20 and 100 nm effectively navigate through lymphatic vessels to reach the lymph nodes.^[^ ^213^, ^214^, ^220^ ^]^ For instance, 20 nm NPs consisting of pluronic‐stabilized polypropylene sulfide (PPS)^[^ ^221^ ^]^ and poly(lactic‐co‐glycolic) acid‐polyethylene glycol (PLGA‐PEG)^[^ ^220^ ^]^ efficiently target the lymph nodes in mice.

Cell‐mediated transport provides an alternative strategy for delivering vaccines to lymph nodes. In this process, peripheral APCs capture cargo at the injection site and migrate via the lymphatic vessels to reach the draining lymph nodes to stimulate T cells in draining lymph nodes.^[^ ^14^ ^]^ Targeting tissue‐resident APCs directly can be accomplished by binding to specific receptors such as C‐type lectin receptors^[^ ^222^ ^]^ (e.g., CD205,^[^ ^223^, ^224^ ^]^ CD40,^[^ ^225^ ^]^ DC‐SIGN^[^ ^226^ ^]^ and MR.^[^ ^227^ ^]^ Particles sized 0.5–1 µm are too large for passive drainage; they can still be transported to lymph nodes by migratory DCs (active transport).^[^ ^214^ ^]^ Conversely, particles exceeding 200 nm may accumulate at the injection site, forming depots that facilitate local infiltration of APCs and prolonged delivery of both antigen and adjuvants (Figure 5c).^[^ ^14^ ^]^

### 4.3 Cellular Barriers

Even with the utilization of NPs in drug delivery systems aimed at targeting specific cells, there remain several obstacles to their uptake and intracellular transportation, which ultimately dictate their functional delivery.^[^ ^205^ ^]^ Anionic NPs might encounter difficulties in reaching the cell surface due to repulsive forces, while excessively charged cationic NPs could potentially damage the cell membrane and cause cytotoxicity.^[^ ^205^, ^228^ ^]^ Upon cellular uptake, NPs undergo a process where endosomes mature and gradually acidify before eventually fusing with lysosomes, organelles responsible for decomposition.^[^ ^229^ ^]^ To activate genetic drugs effectively, they must destabilize the endosome and release the cargo.^[^ ^230^ ^]^ Overcoming the major hurdles can be facilitated by using ionizable substances capable of acquiring a charge within mature endosomes.^[^ ^231^, ^232^ ^]^

Polysaccharide‐based adjuvants have emerged as playing an essential role in overcoming multiple biological and cellular barriers that limit vaccine efficiency. Their unique combination of biodegradability, receptor specificity, and surface adaptability enables efficient transport of antigens across cellular barriers while minimizing systemic toxicity.^[^ ^233^ ^]^ At the cellular interface, through their repeating carbohydrate motifs, polysaccharides promote cellular internalization of antigens by interacting with PRRs expressed on epithelial and immune cells. Structurally diverse polysaccharides such as β‐glucan, mannan, and chitosan engage PRRs, including Dectin‐1,^[^ ^51^, ^234^ ^]^ CD206,^[^ ^235^, ^236^ ^]^ and TLR2/4. This specific molecular recognition initiates receptor‐mediated endocytosis, enabling efficient uptake of polysaccharide–antigen conjugates or polysaccharide‐coated nanoparticles. Unlike nonspecific pinocytic or adsorptive pathways, PRR‐driven internalization directs the cargo to endosomal compartments, where antigen degradation, peptide loading, and innate signaling pathways occur.^[^ ^233^ ^]^ For example, β‐glucan engagement of Dectin‐1 drives the formation of a phagocytic synapse‐like cluster that excludes phosphatases, enabling Syk‐CARD9‐NF‐κB signaling and coordinated cytoskeletal remodeling events that directly promote DC maturation.^[^ ^237^ ^]^ Functionally, Dectin‐1 signaling in DCs induces upregulation of costimulatory molecules (e.g., CD80/CD86) and secretion of IL‐6, TNF, and IL‐23, thereby linking the route of uptake to transcriptional programs that license T‐cell priming.^[^ ^83^ ^]^ In parallel, CD206 on macrophages and DCs mediates high‐affinity endocytosis of mannose‐displaying ligands/particles and participates in antigen processing and presentation, providing a complementary axis to steer antigen into MHC pathways.^[^ ^238^ ^]^ Cationic or partially acetylated polysaccharides such as chitosan can additionally activate DCs through TLR4‐dependent mechanisms, increasing MHC class II expression and co‐stimulation, which reinforces PRR‐guided uptake with pro‐maturational signals.^[^ ^239^ ^]^ Together, these receptor‐specific interactions bias intracellular routing to endosomal/lysosomal compartments that support antigen processing, while simultaneously delivering the danger signals required for efficient antigen presentation and immune priming^[^ ^240^ ^]^ (Figure 5d).

Certain nanoparticle designs incorporate surface modifications, such as PEGylation, self‐derived peptides (including CD47), or cell membrane coatings, to minimize recognition and uptake by phagocytic cells, thereby reducing premature clearance and enhancing systemic persistence.^[^ ^205^ ^]^ PEGylation enhances nanoparticle circulation by modifying size and solubility, while also providing a protective shield against enzymatic degradation and immune recognition. However, this steric barrier is not entirely effective in preventing uptake by macrophages or other immune cells. Moreover, repeated exposure to PEG can lead to the generation of anti‐PEG antibodies, which may accelerate the clearance of PEGylated nanoparticles when present at elevated levels.^[^ ^241^ ^]^

## 5 Application of Polysaccharides in Immune Modulation

### 5.1 Mannan

Mannan is a mannose‐based polysaccharide that can be categorized into the subfamilies: linear mannans, galactomannans, and glucomannans. Linear mannans and galactomannans have a backbone of β(1→4)‐linked _D_‐mannosyl residues, whereas glucomannans has a backbone of β(1→4)‐linked _D_‐mannosyl and D‐glucosyl residues of varying ratios and degrees of polymerization.^[^ ^242^, ^243^, ^244^ ^]^ Mannans can be obtained from a variety of different sources, including fungi (e.g., *Candida albicans*, *Candida glabrata*, and *lichen Xanthoria parientina*), yeasts (e.g., *S. cerevisiae*), plants (e.g., *Konjac*, *Bletilla striata*, and *Sesbania cannabina*), algae, woods, and seeds.^[^ ^245^ ^]^ The MW can vary between 200 and 2000 kDa depending on the source, processing method, and storage time, and the mannose residues in the backbone can be partially substituted by O‐acetyl groups.^[^ ^246^, ^247^ ^]^ In particular, mannan is the prevalent polysaccharide in the outermost layer of yeast cell walls, which can be recognized by TLR‐2, 4, and various CLRs, such as MR, DC‐SIGN, mannose‐binding lectin, and DEC‐205.^[^ ^248^, ^249^, ^250^ ^]^ TLR‐2 or TLR‐4 activation can trigger an inflammatory response via the NF‐kB pathway, stimulate monocytes to produce TNF‐α, and activate inflammasomes and caspase 1 to promote cytokines, such as IL‐1β, IL‐18, and IL‐6. Mannan can also induce a pro‐inflammatory Th17 response by DCs or monocytes with the production of IL‐17, which induces shuttling of polymorphonuclear leukocytes, activation of neutrophils and macrophages, synthesis of antimicrobial peptides, and anti‐tumor responses by CD8^+^ T cells.^[^ ^251^ ^]^ Mannan has a number of immunogenic activities, including tumor growth suppression via inhibition, induction of anti‐oxidative activity and autophagy in tumor cells, activation of macrophages, and targeting and repolarizing tumor‐associated macrophage (TAMs).^[^ ^247^ ^]^

#### 5.1.1 Linear Mannan

Many studies have shown significant uptake of mannosylated delivery systems and subsequent immune regulation by macrophages. Pi et al. decorated mannose on the surface of PEG‐grafted graphene oxide‐based NPs for the targeting and activation of macrophages. When loaded with rifampicin, NPs efficiently killed intracellular *M. tuberculosis* (*Mtb*) bacilli in *Mtb*‐infected macrophages by promoting macrophage activation.^[^ ^252^ ^]^ Pruthi et al. synthesized Amphotericin B (AmB)‐loaded/mannosylated carbon nanotube for sustained release of AmB in macrophage cells and macrophage‐rich organs such as liver and spleen for improving parasiticidal activity of AmB.^[^ ^253^ ^]^ Jain et al. similarly developed mannose‐conjugated poly (propylene imine) dendrimers for the delivery of AmB into macrophages and macrophage‐rich organs without noticeable toxicity.^[^ ^254^ ^]^

Mannan has also been extensively investigated for targeting M2‐type macrophages through the CD206 receptor and repolarizing them into M1‐type macrophages. Wang et al. synthesized mannose‐modified cationic lipid NPs with IMD‐01352 (TAM repolarization agent) and co‐administered with sorafenib (anticancer agent)‐loaded cationic lipid NPs for Hepa1‐6 tumor treatment.^[^ ^49^ ^]^ The co‐delivery system led to a significant decrease in tumor volumes and promoted macrophage repolarization from M2 to M1‐type in vivo following intravenous injection. Song et al. developed mannosylated MnO_2_ NPs that generate oxygen to alleviate immunosuppressive hypoxia in the tumors and increase M1‐type macrophages with hyaluronic acid coating.^[^ ^255^, ^256^ ^]^ Intravenous injection of the NPs combined with doxorubicin (DOX) showed a significant enhancement of chemotherapy response in 4T1 tumor mice. Zhang et al. prepared di‐mannose‐coated mRNA NPs using electrostatic complexation of poly (B‐amino ester) polymer and mRNA‐encoding M1 polarization factors. The NPs effectively targeted and transfected CD206‐positive M2‐type macrophages for M1 differentiation in mice with ID8 ovarian tumors and significantly inhibited local tumors as well as pulmonary B16F10 melanoma metastasis.^[^ ^257^ ^]^ Wang et al. prepared mannosylated lactoferrin NPs containing anti‐cancer shikonin and PD‐L1 suppressor JQ1 as a multi‐target therapy.^[^ ^258^ ^]^ Lactoferrin and mannose promoted targeting of CT26 cancer cells and M2‐type macrophages via low‐density lipoprotein receptor‐related protein 1 and CD206, respectively, which endowed the NPs with robust antitumor effects in mice bearing CT26 colon carcinoma. Zeng et al constructed carboxymethyl chitosan‐NPs and modified them with mannose and imidazole. Intravenous injection of the NPs in mice with Lewis lung carcinoma exhibited specific binding to and apoptosis of M2‐type macrophages in tumors.^[^ ^259^ ^]^

**Figure 6:**
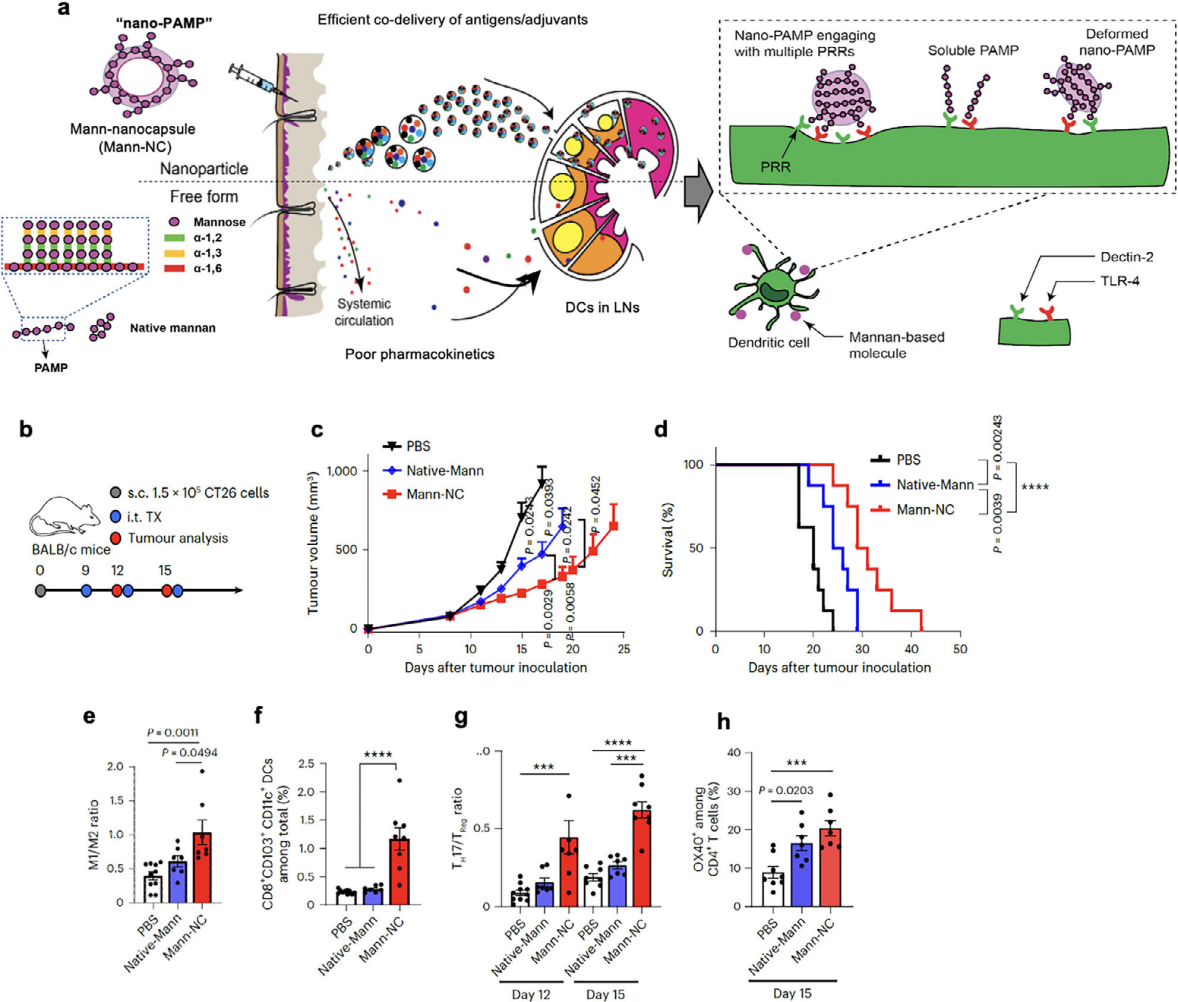
A) Mannan‐based nanocapsules for efficient delivery of antigens or adjuvants to PRR‐expressing DCs in the lymph node. B‐D) Mannan‐based nanocapsules loaded with mOVA showed superior antitumor efficacy in vivo. Experimental timeline: Following intratumoral vaccination with Mann‐NC, tumor growth was delayed and survival improved. E) Mann‐NC increased the M1/M2 macrophage ratio and F) the proportion of CD103^+^CD11c^+^ dendritic cells, indicating innate immune activation and enhanced cross‐presentation. G,H) Adaptive responses were marked by elevated OX40^+^CD4^+^T cells and a higher Th1/Treg ratio, indicating a shift toward anti‐tumor T cell immunity. Reproduced with permission.^[^ ^133^ ^]^ Copyright 2023, Springer Nature.

Mannan has been widely used as a main component of vaccines against cancers or infections. Rezaei et al. prepared iron oxide NPs and surface‐loaded Hepatitis B surface antigen and mannose and showed that mannose promoted expression of pro‐inflammatory cytokines and lymphocyte proliferation with an increased IgG2a/IgG1 ratio.^[^ ^260^ ^]^ Liu et al. demonstrated that mannan can protect mice from the infection of A. fumigatus conidia with bovine serum albumin (BSA) conjugation, improving the efficiency by 40‐fold in a dose‐dependent manner.^[^ ^261^ ^]^ Proudfoot et al. conjugated oxidized mannan to whole inactivated hemagglutinin type 1 and neuraminidase type 1 (H1N1) influenza virus and assessed serum and mucosal immunity after intranasal administration. The vaccine induced serum IgG1 and IgG2a and inhibited H1N1‐mediated red blood cell agglutination.^[^ ^262^ ^]^ In a notable approach of polysaccharide engineering by Son et al., a rational design of polysaccharide‐based NPs has been suggested to facilitate multivalent PAMP‐PRR interaction to promote immune responses. Linear mannan was engineered to form a unique, hollow nanocapsule 200 nm in diameter with a desirable surface mannan PAMP arrangement for better PRR engagement, mimicking the mechanism by which naturally occurring pathogens engage multiple PRRs by their particulate structure (**Figure** **6**a‐h). Upon subcutaneous injection, the mannan nanocapsules were efficiently drained to the lymph nodes and highly taken up by DCs and macrophages. Nanocapsules interacted with Dectin‐2 and TLR4 to induce TH17 cell differentiation, leading to decreased Tregs and increased TH17, CD8^+^ T cells, NK cells, and M1 macrophages in tumors, while OX40 blockade further boosted antitumor effects.^[^ ^133^ ^]^ The mannan capsules loaded with ovalbumin (OVA)‐encoding mRNA led to significant bone marrow‐derived dendritic cell (BMDC) activation via Dectin‐2 and TLR‐4 to promote antigen presentation for the induction of strong antigen‐specific CD4^+^ and CD8^+^ T cell responses with robust anti‐tumor efficacy in a murine model of B16 cells expressing OVA.^[^ ^263^ ^]^

Additionally, Medina et al. introduced a combination of mannan, TLR ligands, and agonistic anti‐CD40 monoclonal antibody (MBTA) to modulate the immune phenotype of injected tumors. Intratumor or subcutaneous injection of MBTA as a whole tumor cell vaccine resulted in an increased CD8^+^ T cell tumor infiltration. MBTA was discovered to enhance phagocytosis of tumor cells and regulate tumor‐specific adaptive immune responses by modulating neutrophil and APC trafficking to tumors.^[^ ^264^ ^]^

Mannan has also shown a variety of other immunogenic effects, including proliferation of NK cells, suppression of LPS‐induced macrophage activation, and downregulation of immune inhibitory receptor PD‐L1 on myeloid‐derived suppressor cells (MDSCs). Yang et al. orally administered D. tosaense‐derived polysaccharide composed of galactose:glucose:mannose (1:9.1:150.7) and demonstrated the activation and proliferation of splenic T cells and NK cells, possibly due to the interactions with microfold cells in the intestine, which transport molecules in the Peyer's patches.^[^ ^265^, ^266^ ^]^ Torretta et al. showed _D_‐mannose suppressed LPS‐induced macrophage activation by impairing IL‐1β production, highlighting its potential to treat inflammatory conditions.^[^ ^267^ ^]^ Kempe et al. synthesized hollow glycopolymer microcapsules composed of poly(oligo(2‐ethyl‐2‐oxazoline methacrylate)‐stat‐glycidyl methacrylate) and mannose azide and investigated their interaction with APCs, which revealed that the microcapsules induced cytokine‐independent direct upregulation of CD80 on DCs and indirect downregulation of PD‐L1 on MDSCs.^[^ ^268^ ^]^ Yoon and Kang et al. synthesized mannan‐decorated inulin acetate microparticles (M‐IA MPs) as carriers and adjuvants for recombinant foot‐and‐mouth disease multi‐epitope subunit vaccine (M5BT). These microparticles are efficiently internalized by APCs such as macrophages and DCs via phagocytosis. M‐IA MPs were demonstrated to boost host immune response in vivo through production of M5BT‐specific antibodies and anti‐FMDV (Foot‐and‐mouth disease virus) antibodies, alongside promoting Th1 responses.^[^ ^269^ ^]^

#### 5.1.2 Glucomannan

It has been shown that glucomannans exhibit immunostimulatory activity through TLR‐2 or TLR‐4 signaling pathways. Huang et al. examined the effects of 2,3‐*O*‐acetylated‐1,4‐*β*‐d‐glucomannan on human leukemia monocytic cells and observed activation of the TLR‐4 signaling pathway associated with the expression of C‐C motif chemokine ligand 4 (CCL4) and CXCL10.^[^ ^270^ ^]^ Feng et al. investigated the acetylation of glucomannan for macrophage‐regulatory activity, such as TLR‐2 activation, M1‐type expression, and release of proinflammatory cytokines, which was found to be heavily dependent on the degree of acetylation, sugar unit, and size of the NPs.^[^ ^136^ ^]^

Glucomannans have been utilized in macrophage‐targeting cancer therapy and vaccine applications. Zhan et al. synthesized an alendronate(ALN)‐glucomannan conjugate that can target and eliminate TAMs via glucomannan and ALN, respectively.^[^ ^271^ ^]^ Spontaneously formed ALN‐BSP (polysaccharide from *Bletilla striata)* NPs inhibited angiogenesis and tumor growth and prolonged animal survival with reduction of TAMs and activation of cytotoxic lymphocytes in S180 sarcoma‐bearing mice. The same group further developed ALN‐BSP NPs to improve their systemic circulation and tumor targeting with a bio‐responsive polymeric shell consisting of PEG, poly(lactic‐co‐glycolic acid), and MMP‐cleavable peptide.^[^ ^272^ ^]^ Konjac glucomannan has been most widely studied for vaccine applications due to its high biocompatibility, biodegradability, and biological activity.^[^ ^273^, ^274^ ^]^ Shi et al. synthesized NPs with carboxymethyl konjac glucomannan (CKGM) and 2‐hydroxypropyl trimethyl ammonium chloride chitosan and developed the NPs as a biocompatible nanovaccine platform by encapsulating OVA.^[^ ^274^ ^]^ In a follow‐up study, the same group synthesized OVA‐loaded NPs using anionic CKGM and cationic quaternized konjac glucomannan and demonstrated robust cellular immune responses characterized by IL‐2 and IFN‐γ production.^[^ ^275^ ^]^

Other glucomannans have also been actively investigated for drug delivery applications. Dong et al. introduced ethylenediamine to a glucomannan derived from *Bletilla striata* and synthesized a glucomannan/DNA nanocomplex via electrostatic interaction. The nanocomplex resulted in significantly higher transfection efficiency in RAW 264.7 macrophages than conventional gene transfection agents based on polyethyleneimine (PEI) or lipofectamine, which was ascribed to CD206 receptor binding.^[^ ^235^ ^]^ Harde et al. developed glucomannosylated chitosan NPs and encapsulated BSA as a model antigen for demonstrating oral vaccine delivery.^[^ ^38^ ^]^ The NPs were efficiently internalized into RAW 264.7 macrophages by mannose and glucose transporter‐mediated endocytosis, and the glucomannan modification induced high levels of serum IgG, secretory IgA, and IL‐2 and IFN‐γ when orally administered to male BALB/c mice, indicating broad‐spectrum humoral, mucosal, and cell‐mediated immune responses.

#### 5.1.3 Galactomannan

Galactomannans exhibit varying mannose/galactose ratios from 1.1 to 5.0, where the high galactose contents generally increase the water solubility, functionality, and activity of galactomannans.^[^ ^276^ ^]^ The immunological properties of galactomannans have been actively studied. Ha et al. showed that galactomannan conjugated to lysozyme can promote production of NO, TNF‐α, IL‐1β, and IL‐8 by macrophages via MAPK and NF‐kB signaling pathways.^[^ ^277^ ^]^ Li et al. extracted a galactomannan from *Sesbania cannabina* and showed that it exhibited dose‐dependent radical scavenging and ferrous ion chelating activity, as well as macrophage stimulation and cytokine induction via TLR‐2 and TLR‐4 pathways.^[^ ^278^ ^]^ Perera et al. showed that galactomannan isolated from *Antrodia cinnamomea* promoted TNF‐α release, phagocytosis, and bactericidal activity, and endotoxin tolerance‐like effect of macrophages via TLR‐4 interaction.^[^ ^279^, ^280^ ^]^ Rashid et al. investigated highly branched galactomannan obtained from lichen *Xanthoria parientina* for dose‐dependent stimulation of IL‐1β, TNF‐α, and phagocytosis by macrophages through the interaction with Dectin‐2.^[^ ^281^ ^]^ Galactomannan from *Punica granatum* exhibited anti‐angiogenic and anti‐metastatic properties mainly by inhibiting the activation of matrix metalloproteinase and up‐regulating tissue inhibitors of metalloproteinases.^[^ ^282^ ^]^ Kumari et al. prepared PEGylated galactomannan NPs loaded with hydrazinocurcumin and showed that the NPs repolarized M2‐type macrophages towards M1‐type in vitro and inhibited the growth of Ehrlich's ascites carcinoma in mice.^[^ ^283^ ^]^ Toledano et al. showed that galactomannan induced a reprogramming of the inflammatory response by human macrophages through the Dectin‐1 and NF‐kB signaling, which significantly alleviated LPS‐induced inflammation.^[^ ^284^ ^]^

Galactomannan has also been developed for drug delivery and cancer therapy. Padinjarathil et al. synthesized silver NPs using galactomannan from *Punica granatum* as a reducing as well as capping agent. The NPs induced selective cytotoxicity against human cancer cell lines of A549 (adenocarcinoma alveolar basal epithelial cells), HCT116 (colorectal carcinoma cells), HepG2 (hepatocellular carcinoma cells) in vitro via caspase‐mediated apoptotic pathways, demonstrating their potential for cancer treatment.^[^ ^285^ ^]^ Cerqueira et al. developed galactomannan as a carrier of hydrophobic drugs and showed that acetylated galactomannan micelles efficiently encapsulated curcumin.^[^ ^286^ ^]^ Zhang et al. prepared NPs with glycogen, polysaccharide‐binding concanavalin A, and galactomannan for the delivery of DOX to liver cells. The galactose residues of galactomannan allowed targeted delivery via the liver‐specific asialoglycoprotein receptor, leading to enhanced cytotoxicity toward human liver cells.^[^ ^287^ ^]^ Zaritski et al. designed galactomannan‐based self‐assembled amphiphilic NPs that can target tumors expressing glucose transporter‐1, a transmembrane protein responsible for the transport of glucose, mannose, and lower‐affinity galactose. The intratumoral accumulation of the NPs was well correlated with glucose transporter‐1‐expression by cancer cells in murine Rhabdomyosarcoma and Ewing sarcoma tumor models.^[^ ^288^ ^]^ Moretton et al. developed flower‐like polymeric micelles (PMs) coated with chitosan and hydrolyzed galactomannan (GalM‐h) for delivering rifampicin to macrophages. The PMs exhibited highly efficient encapsulation of rifampicin and GalM‐h residues, which allowed for a significant increase in macrophage uptake of rifampicin‐loaded PMs.^[^ ^289^ ^]^

### 5.2 Glucan

Glucans are the most abundant polysaccharides found in the inner cell walls of bacteria, fungi, yeast, and plants, which are mainly composed of _D_‐glucose monomers linked via glycosidic bonds.^[^ ^290^, ^291^ ^]^ In particular, β‐glucan is a homopolymer of glucose with linear (1→3)‐β‐_D_‐glycosidic linkages or branched side chains bound by (1→6)‐β‐_D_‐glycosidic linkages. β‐glucans can be obtained from various sources, including Curdlan (*Alcaligenes faecalis*), laminaran (*Laminaria sp*.), Schizophyllan (*Schizophyllum commune*), and yeast (*Saccharomyces cerevisiae*), which exhibit unique physicochemical and biological activity depending on the sources and chemical modifications. For example, β‐glucans can be categorized as soluble and gel‐forming β‐glucans of a high‐molecular branched form (e.g., schizophyllan) or a linear form (laminaran from brown sea algae), insoluble particulate β‐glucans of a blended high‐molecular linear and branched form, or chemically modified particulate β‐glucans with carboxymethylated, sulfonated, or phosphorylated forms.^[^ ^292^ ^]^ β‐glucan is known as a potent immune modulator that acts on various innate immune cells, with macrophages being the primary target cells. PRRs and other receptors, including Dectin‐1, TLR‐2, and CR3, can recognize β‐glucan and promote its phagocytosis and lysis. As a result, β‐glucan triggers a range of immune responses, including the release of cytokines and chemokines like IL‐1, IL‐9, and TNF‐α, upregulation of adhesion molecules, and proliferation of T‐cells.^[^ ^293^, ^294^, ^295^, ^296^, ^297^, ^298^ ^]^

β‐glucans have been developed as immune‐stimulating delivery platforms for cancer therapy. Maeda et al. synthesized a self‐assembled nanogel using naphthalene‐modified β‐1,3‐glucan extracted from the fungus *Schizophyllum Commune*.^[^ ^299^ ^]^ The nanogel was further loaded with DOX, which showed high cytotoxicity to mouse leukemic monocyte macrophages via Dectin‐1‐mediated cellular uptake. Curdlan is a bacteria‐derived water‐insoluble linear β‐glucan exclusively composed of (1,3)‐β‐glucosidic linkages.^[^ ^300^, ^301^ ^]^ In a study conducted by Nasrollahi et al., oxidized curdlan was fabricated into NPs by conjugating trastuzumab antibodies and DOX, followed by PEI coating to selectively deliver DOX to human epidermal growth factor receptor 2 (Her2)^+^ breast cancer. The curdlan/DOX NPs induced efficient tumoricidal effects with tumor growth suppression and extended mice survival in a 4T1 breast cancer model.^[^ ^15^, ^302^ ^]^ Carboxymethylation can improve the solubility and physicochemical properties of β‐glucan in aqueous solution.^[^ ^303^, ^304^ ^]^ Intraperitoneal injection of carboxymethyl glucan together with cyclophosphamide significantly inhibited intramuscular Lewis lung carcinoma by eliciting an anti‐tumor effect of macrophages along with cyclophosphamide chemotherapy.^[^ ^304^ ^]^ In another study, β‐glucan was conjugated with the MUC1 tandem repeat sequence and injected subcutaneously into mice bearing MCF‐7 tumors for cancer vaccination. The conjugate group produced high levels of anti‐MUC1 IgG antibodies and significantly inhibited MCF‐7 tumor growth.^[^ ^305^ ^]^ In a study conducted by Soto et al., mesoporous silica NPs (MSNs) were loaded with DOX and adsorbed onto PEI‐modified glucan microparticles with a diameter of 2‐4 µm via electrostatic interaction. The resulting Dox‐MSN‐PEI‐GPs (glucan particles) efficiently targeted NIH 3T3‐D1 cancer cells through Dectin‐1 and CR3 and inhibited tumor growth at a DOX concentration level below an effective free‐drug concentration. β‐glucan has also been used for siRNA delivery. Zhang et al. reported a NP formulation of siRNA using 6‐amino‐6‐deoxy‐curdlan (6AC),^[^ ^306^ ^]^ where 6AC with 100% amine substitution NPs efficiently formed a nanocomplex with siRNA. 6AC NPs promoted siRNA delivery to cancer cells, primary mouse cells, and mouse stem cells, and their therapeutic efficacy was demonstrated in a mouse 4T1 tumor model using siRNAs against tumor‐promoting migration inhibitory factor.^[^ ^307^ ^]^ Li et al developed a biphasic delivery system incorporating PLGA NPs encapsulating the trained immunity inducer muramyl dipeptide (MDP) and specific tumor antigen human papillomavirus E7 peptide. These NPs were intended to be delivered in combination with β‐glucan in a sodium alginate hydrogel. β‐glucan was shown to enhance the encapsulated antigen uptake by the APCs, and it facilitates DC maturation, potentially through phosphoinositide 3‐kinase (PI3K)/Akt signaling pathway, as indicated by the increased expression of CD80, CD86, MHC II, and MHC I. Additionally, in vitro studies using peripheral blood mononuclear cells demonstrated increased proinflammatory cytokines due to heterologous secondary stimulation, indicating the memory effects of trained innate immunity.^[^ ^308^ ^]^ Wang et al utilized Whole Glucan Particle (WGP) β‐glucan as an immune adjuvant together with programmed cell death protein 1 (PD‐1)/PD‐L1 to decrease resistance to immunotherapy. This combination treatment inhibited tumor progression in a murine Lewis lung carcinoma model compared to each treatment administered alone. WGP facilitated infiltration of DCs and macrophages into the tumor microenvironment while reshaping its suppressive nature by decreasing regulatory T cells and MDSCs. Additionally, WGP led to an elevated concentration of proinflammatory cytokines such as IL‐1β, IL‐6, and IL‐8, known to increase the expression of inhibitory immune checkpoints such as PD‐L1.^[^ ^309^ ^]^ Mao et al developed a self‐assembled nanodrug, lentinan (LNT) and ursolic acid, for the regulation of immunosuppressive TME, resulting in the inhibition of tumor progression in the CT26 CRC tumor model through mobilization of both innate and adaptive immunity. LNT as an immune adjuvant promotes DC maturation, TAM polarization towards antitumor M1 phenotype, effector T cell recruitment, and production of antitumor‐related cytokines such as IFN‐γ, TNF‐α.^[^ ^310^ ^]^

Glucan particles have attracted great interest for preventing bacterial infections via macrophages.^[^ ^311^ ^]^ Basha et al. developed cyclodextrin‐conjugated curdlan NPs for the targeted delivery of two tuberculosis drugs, rifampicin and levofloxacin, into the bacteria‐infected macrophages. The NPs demonstrated selectivity toward macrophages over fibroblast cells by Dectin‐1‐mediated targeting, which improves bactericidal activity of rifampicin and levofloxacin in M. smegmatis‐infected mouse macrophage cells.^[^ ^312^ ^]^ In another study, yeast‐derived glucan microparticles were prepared by spray drying and used for the delivery of Rifabutin NPs to the macrophages. Rifabutin NP‐encapsulating glucan microparticles exhibited improved bactericidal effects compared to empty Glucan particles or soluble RB in J774A.1 mouse macrophage cell infected with M. tuberculosis.

**Figure 7:**
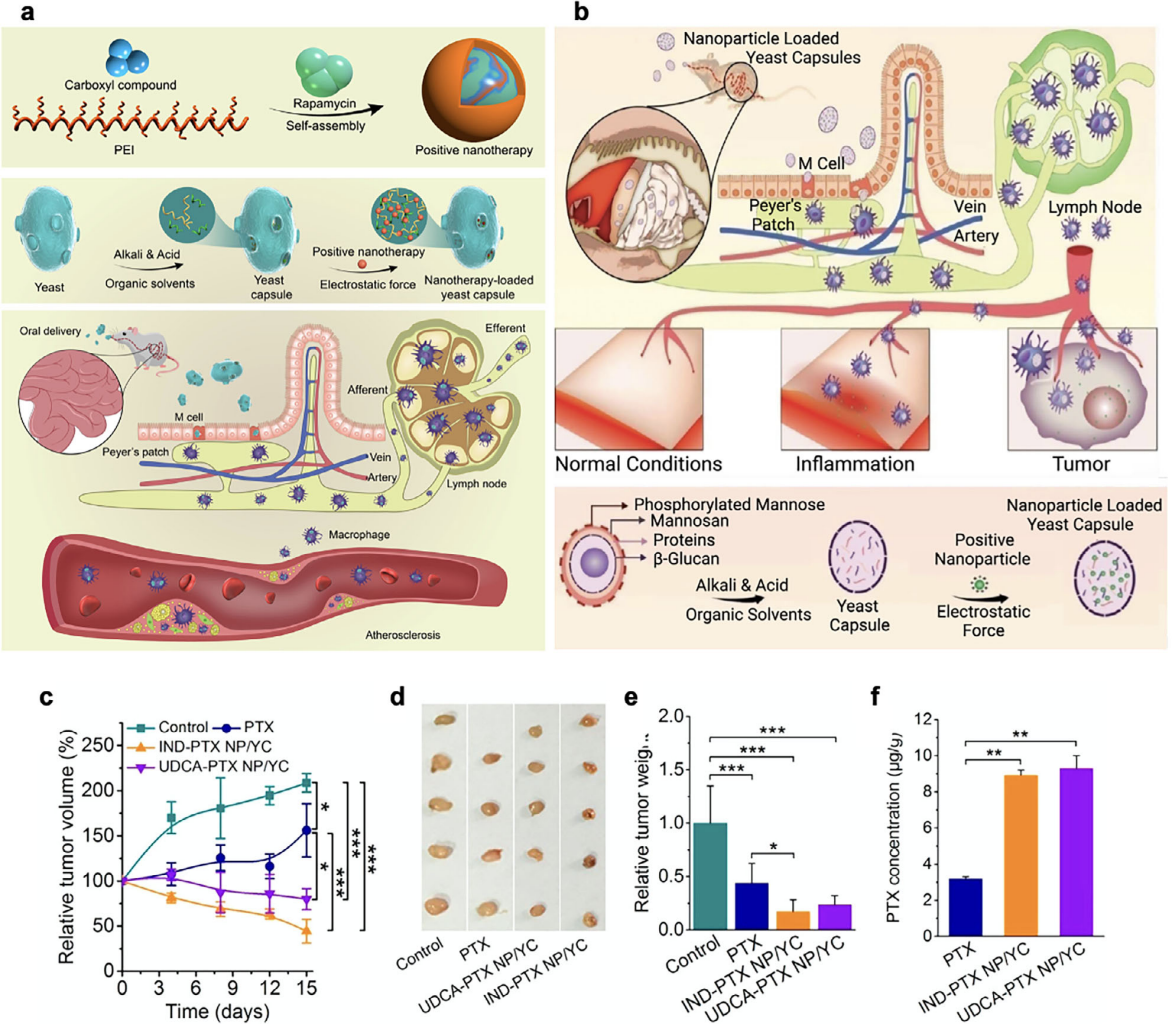
A) Self‐assembled positive nanotherapies were packaged into yeast capsules (YC), and following oral administration, they were delivered to the targeted atherosclerotic plaques by macrophage‐mediated translocation. With the surface of the YC composed mainly of β‐glucan and the β‐glucan receptor, Dectin‐1, expressed on M cells and macrophages, this targeted transportation was likely due to enhanced transepithelial absorption by M cells in Peyer's patches and consequent cellular translocation by monocytes or macrophages through the lymphatic system. Reproduced with permission.^[^ ^51^ ^]^ Copyright 2017, Elsevier. B) YCs loaded with nanotherapies were also used to target and deliver to tumors distant from the GI tract. C–F) Mice bearing MCF‐7 xenografts were treated with indomethacin (IND)‐paclitaxel (PTX) NP/YC or ursodeoxycholic acid (UDCA)‐PTX NP/YC, leading to C) reduced tumor growth, D) noticeably smaller sizes of excised tumors, E) reduced tumor weight, and F) higher PTX concentrations in excised tumors. Reproduced with permission.^[^ ^234^ ^]^ Copyright 2017 American Chemical Society.

For the treatment of cardiovascular disease, yeast glucan capsules were co‐loaded with drugs of anti‐inflammatory Indomethacin and anti‐atherosclerotic Rapamycin in the form of their co‐assembly NPs (**Figure** **7**a).^[^ ^51^ ^]^ When administered into ApoE^−/−^ mice via oral gavage, these NPs accumulated in macrophages by transcytotic absorption in the GI tract via M cells in Peyer's patches, retained up to 7 days, and suppressed macrophage migration and translocation to inflammation sites (Figure 7b‐f).^[^ ^234^ ^]^

### 5.3 Alginate

Alginate is a main component of the cell walls of brown algae, including *Ascophyllum nodosum, Laminaria hyperborea*, and *Macrocystis pyrifera*, as well as of many other bacteria. Alginate is a polymeric form of 1‐4 linked β‐_D_‐mannuronic acid (M‐block) and α‐_L_‐guluronic acid (G‐block) units repeating in either a homogenous (consecutive M or G residues) or heterogeneous (alternating M‐G residues) pattern. The proportion of M and G blocks is an important determinant for the biological and physical properties of alginates. For example, alginates of high M/G block ratios promote macrophages to secrete various pro‐inflammatory cytokines, including IL‐1, IL‐6, IL‐12, and TNF‐α through interactions with the monocyte CD14 receptor.^[^ ^37^ ^]^ On the other hand, G‐block provides the coordination sites with multivalent cations such as Ca^2+^ ions for cross‐linked gel formation. Alginate can also elicit adaptive immune responses associated with the activation of CD8^+^ T cells and the development of CD44^+^CD62L^+^ central memory T cells by cellular anti‐oxidation function.^[^ ^313^ ^]^ Alginate has attracted great interest for biomedical applications, particularly in the form of a lyophilized porous three‐dimensional (3D) network structure or hydrogel scaffold. The mechanical properties of alginate gels can be controlled by the G‐block length and total molecular weight, whereas the release of cargo molecules bound on or encapsulated in the scaffold can be controlled by pH or other stimuli.^[^ ^314^ ^]^ U.S. Food and Drug Administration (FDA) approved alginate for a number of different biomedical applications, including regeneration medicine and nutrition supplements.^[^ ^315^ ^]^

One of the most extensively investigated developments of alginate is for cancer therapy using the immunostimulatory and sustained cargo release property of alginate gel. Xiong et al. fabricated an alginate hydrogel encapsulating X‐C motif chemokine ligand 1 (XCL‐1) via in situ gelation.^[^ ^50^ ^]^ XCL‐1 is a chemokine involved in the recruitment of DCs and their uptake and cross‐presentation of antigens for the priming of T cells. Alginate formed an in situ gel at the physiological Ca^2+^ concentration in tumors, and the release of XCL‐1 chemokines attracted XCR‐1^+^ DCs into tumors, promoting cross‐presentation of tumor‐associated antigens by the tumor‐infiltrated DCs. Consequently, it was shown to improve the anti‐tumor efficacy of co‐administered DOX‐loaded PLGA NPs by augmenting an anti‐tumor immune response triggered by DOX‐mediated immunogenic cell death. Yan et al. prepared a hybrid alginate/collagen composite gel using charge‐based self‐assembly of alginate and collagen, which was utilized for the delivery of methylene blue and imiquimod as a photothermal agent and immune adjuvant, respectively.^[^ ^316^ ^]^ Intratumoral injection of the composite gel, followed by laser irradiation, not only eradicated 4T1 tumors by local heating but also inhibited tumor recurrence and metastasis by augmenting antitumor immune responses of photothermal therapy. In another study, alginate was chemically conjugated with OVA and DC‐targeting mannose ligands, followed by Ca^2+^‐dependent cross‐linked NP formation for cancer vaccine application.^[^ ^317^ ^]^ The alginate nanovaccine promoted the secretion of cytokines and upregulation of co‐stimulatory surface markers by BMDCs in vitro, and led to significant antitumor effects and tumor growth inhibition associated with antigen‐specific cytotoxic T lymphocyte response in a mouse E.G7 tumor model. As such, alginate has received great attention as a vaccine delivery platform. Sinha et al. developed a 3D macroporous alginate scaffold embedded with reduced graphene oxide (rGO) for high loading and slow release of cargo molecules with the hydrophobic surface and large surface area of rGO.^[^ ^318^ ^]^ Subcutaneously implanted alginate‐rGO scaffold attracted a large number of DCs and CD4^+^/CD8^+^ memory T cells, and resulted in the significant inhibition of B16‐OVA tumor growth with the loading of OVA, granulocyte‐macrophage colony‐stimulating factor (GM‐CSF), and cytidine–phosphate–guanosine (CpG). In another study, injectable methacrylated algihnate cryogels were created by a double‐crosslinking strategy associated with the sequential treatment of radical polymerization and Ca^2+^‐mediated ionic crosslinking.^[^ ^319^ ^]^ The alginate cryogels loaded with OVA, GM‐CSF, and CpG effectively recruited and activated DCs and induced strong OVA‐specific cytotoxic T‐lymphocyte and humoral responses, leading to strong protective immunity against HER2/neu‐overexpressing breast cancer cells. The injectable alginate cryogels were further employed in combined chemoimmunotherapy using Nutlin‐3a, which elicits immunogenic cell death, specifically killing wild‐type tumor protein p53 (p53) cancer cells by activating the p53 tumor suppressor gene.^[^ ^320^ ^]^ The alginate cryogels were loaded with GM‐CSF and CpG, and further combined with Nutlin‐3a‐loaded, spermine‐modified acetalated dextran NPs. The resulting injectable composite gel demonstrated an anti‐proliferation effect against p53‐positive EL4 cancer cells with Nutlin‐3a‐mediated immunogenic cell death. For the advanced mechanical engineering of alginate gel, stimuli‐responsive crosslinking strategies have been employed as an alternative to Ca^2+^‐mediated non‐responsive ionic crosslinking. For example, bio‐reducible cationic alginate‐PEI nanogels were prepared using the electrostatic interaction between alginate and PEI, followed by reduction‐sensitive disulfide crosslinking of PEI residues.^[^ ^321^ ^]^ The bioreducible alginate‐PEI nanogel enhanced intracellular processing and MHC class I/ II presentation of antigens compared to a non‐bioreducible nanogel, leading to robust OVA‐specific humoral and cellular immune responses in vivo after intraperitoneal injection. Similarly, a biodegradable alginate‐chitosan gel scaffold was fabricated using a pH‐responsive Schiff‐base reaction between oxidized alginate and *N*‐succinyl chitosan.^[^ ^322^ ^]^ The pH‐responsive composite gel was developed for mRNA vaccine delivery, in which OVA mRNA lipoplex‐loaded gel scaffolds elicited OVA‐specific cellular and humoral immune responses to a greater extent than naked mRNA or OVA mRNA lipoplex controls.

Alginate has also been tested for preventing bacterial infections via macrophages. Vaghasiya et al. prepared calcium alginate microspheres with a spray‐congealing process and examined their bacillary‐killing effect against *Escherichia coli*‐infected THP‐1‐derived macrophage cells.^[^ ^48^ ^]^ Alginate microspheres were more efficiently and rapidly phagocytized by the infected macrophages than by normal macrophage cells, and subsequently promoted the secretion of pro‐inflammatory cytokines and the generation of ROS and NO in the infected cells. Such M1‐like inflammatory response in the infected macrophages was associated with the anti‐bacterial activity, limiting *Escherichia coli* replication and the total bacillary burden inside the cells. Alginate was also employed for the surface coating of chitosan NPs that encapsulate CpG and proline‐proline‐glutamic acid 17 protein as an immune adjuvant and T cell antigen of Mycobacterium tuberculosis, respectively.^[^ ^44^ ^]^ Chitosan served as a mucoadhesive immune modulator and alginate as a biodegradable and biocompatible protection layer for effective Tuberculosis vaccine application. After primary intranasal administration, the core‐shell NPs induced Th1 and Th17 immune response characterized by the secretion of IFN‐γ and IL‐17 cytokines. A similar or slightly higher immune response was observed after a boost injection of the NPs via subcutaneous administration, which demonstrated the bilateral potency of the core‐shell NPs as booster and prime vaccines upon subcutaneous and intranasal administration. Alginate is also associated with viruses due to its anti‐inflammatory properties. Qin et al developed an oral microencapsulation consisting of inactivated porcine epidemic diarrhea virus, sodium alginate, and chitosan to improve oral delivery efficacy in mice. Alginate and chitosan, in synergy, inhibited proinflammatory cytokines such as IL‐1, TNF‐α, and IL‐17 while improving the viability of DCs and B cells.^[^ ^323^ ^]^

### 5.4 Hyaluronic Acid

Hyaluronic acid (HA) is a non‐sulfated anionic glycosaminoglycan with _D_‐glucuronic acid and N‐acetyl‐_D_‐glucosamine repeating units linked via alternating β (1→3) and β (1→4) glycosidic bonds. HA is the main component of the ECM in the connective, endothelial, and neural tissues and plays an important role in the inflammatory cascade at the site of wound and tissue damage.^[^ ^324^ ^]^ TLR‐2, TLR‐4, and NLRP3 are involved in HA recognition and induction of early innate and adaptive immune responses associated with tissue inflammation. In addition, HA exhibits various biological activities with strong binding to CD44 receptors widely expressed on normal cells, immune cells, and even cancer cells.^[^ ^325^, ^326^ ^]^ The CD44‐binding affinity of HA can be regulated by physicochemical properties like MW. For example, NPs coated with high‐MW HA exhibited greater affinity than the NPs with low‐MW HA.^[^ ^327^ ^]^ Of note, low‐MW HA showed immune‐modulating effects by promoting NF‐κB transcription and pro‐inflammatory cytokines via CD44 and TLR‐2/4 interactions.^[^ ^328^ ^]^ Transdermal administration can take advantage of the unique features of the skin for immune modulation, such as an abundance of tissue‐resident immune cells and direct access to connected organs, avoiding first‐pass metabolism.^[^ ^329^ ^]^ In particular, HA has attracted great attention for topical/transdermal applications as it has demonstrated efficient skin permeation via skin hydration and strong interactions with the stratum corneum and skin cells.^[^ ^330^ ^]^ For example, Kim et al. prepared OVA‐conjugated a HA‐methacrylate and schizophyllan‐methacrylate hybrid nanogel for transdermal immune modulation.^[^ ^331^ ^]^ The hydrogel nanogel promoted skin penetration for deposition in the dermis and cellular uptake by DCs, leading to robust activation and maturation of DCs. The conjugation of various molecules and biologics to the carboxyl group of the _D_‐glucuronic acid unit and/or the hydroxyl group of the N‐acetyl‐_D_‐glucosamine unit of HA allows chemical and structural engineering to promote HA's biological performance and activity.

**Figure 8:**
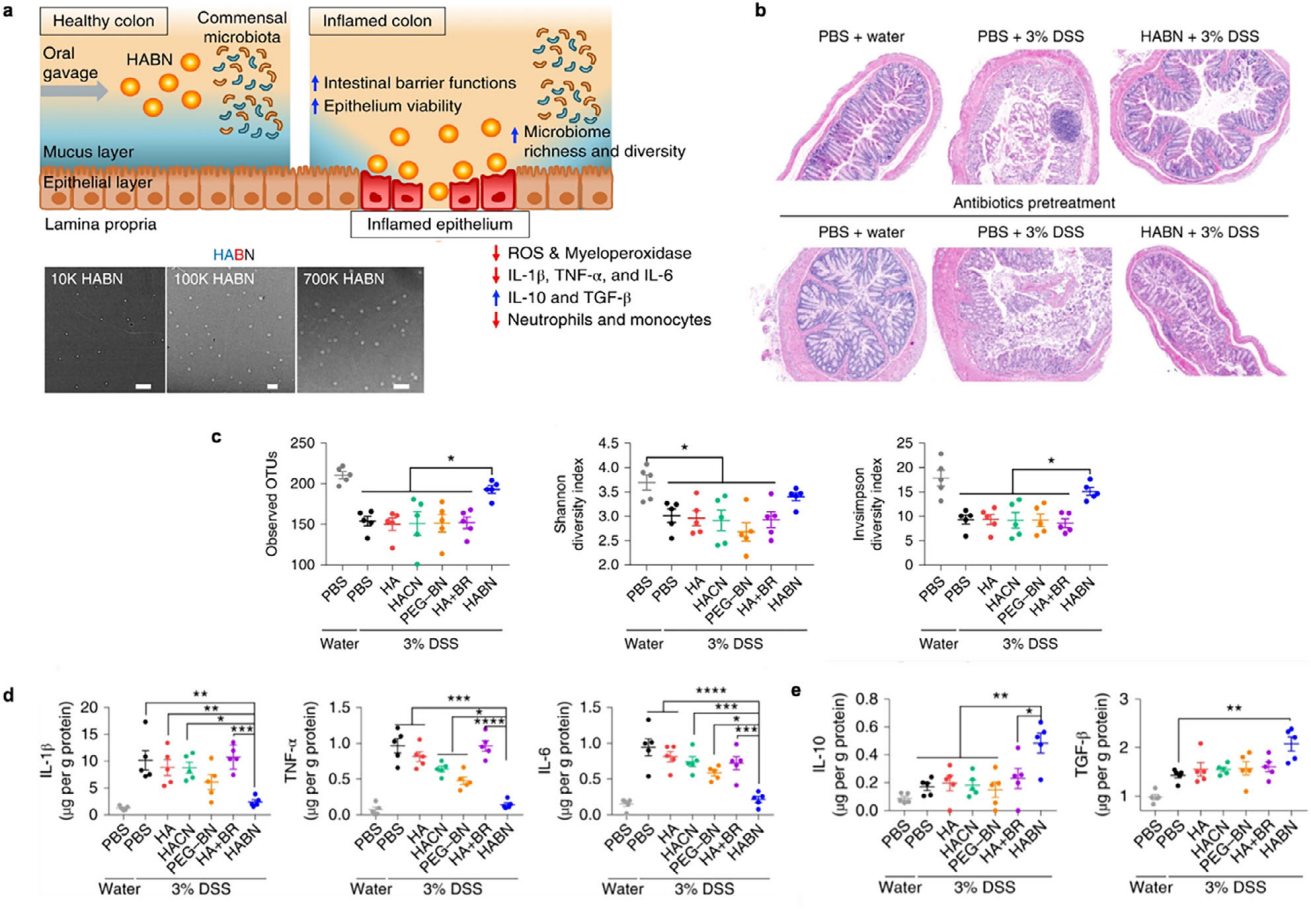
HABN design enables microbiota preservation, inflammation suppression, and mucosal protection during DSS‐induced colitis. A) Schematic of HABN nanoparticles formed by self‐assembly of HA–BR conjugates, along with representative TEM images. HABN preferentially accumulates in the inflamed colon and provides therapeutic benefits in acute colitis. Scale bars: 500 nm (10K HABN), 300 nm (100K HABN), and 400 nm (700K HABN). B) HABN treatment significantly reduces colonic tissue damage and preserves histological architecture in DSS‐challenged mice. C) HABN maintains higher microbial richness and α‐diversity (observed OTUs, Shannon, and inverse Simpson indices) compared to other DSS‐treated groups. D, E) Cytokine profiling shows that HABN increases anti‐inflammatory cytokines (IL‐10, TGF‐β) while reducing pro‐inflammatory mediators (IL‐6, TNF‐α, IL‐1β), supporting mucosal healing and immune regulation. Reproduced with permission.^[^ ^53^ ^]^ Copyright 2020, Springer Nature.

HA has been employed for cancer therapy using its immunostimulatory and CD44‐targeting properties. Miyazaki et al. synthesized a liposome using 2‐carboxycyclohexane‐1‐carboxylated HA and encapsulated OVA, which induced a Th1‐biased anti‐tumor immune response and anti‐tumor effect in mice bearing D.G7‐OVA tumors.^[^ ^332^ ^]^ Yin et al reported that HA can synergize with paclitaxel to inhibit the growth of U14 cervical tumor by promoting the expression of vitamin D3‐binding proteins and the activation of macrophages.^[^ ^333^ ^]^ In another study, a cationic 1,2‐dioleoyl‐3‐trimethylammonium propane (DOTAP) liposome was incorporated with MPLA and OVA and further surface‐modified with PEG and HA for cancer vaccination against OVA.^[^ ^334^ ^]^ The liposome induced maturation and priming of DCs and CD8^+^ T cells, and promoted the generation of OVA‐specific IgG_1_ for effective cancer treatment. HA‐coated cationic NPs can promote endocytosis and subsequent endosomal escape via CD44 receptor interaction and the proton sponge effect.^[^ ^335^, ^336^ ^]^ Similarly, it was reported that HA surface coating can improve DC‐targeted delivery and endocytosis of DOTAP‐PLGA, leading to robust activation of DCs, CD4 T cells, and CD8 T cells for cancer vaccination.^[^ ^337^ ^]^ In addition, HA has been tested for the delivery of multiple anti‐cancer drugs (e.g., irinotecan, DOX, 5‐fluorouracil, and methotrexate) in clinical trials.^[^ ^338^ ^]^ A subset of these preclinical data suggests moderate anticancer efficacy and improved safety profiles. For example, a phase 1 clinical trial with 12 patients indicated that HA‐irinotecan is safe, well‐tolerated, and does not compromise the anticancer activity of irinotecan.^[^ ^339^ ^]^ Another phase 2 trial with 41 patients highlighted the advantages of HA nanoformulations for targeting CD44 isoforms created by variable exon splicing and post‐transcriptional modifications in gene expression.^[^ ^340^ ^]^ Pramanik et al demonstrated the promising cancer therapeutic potential of HA‐functionalized cubosomes, liquid crystalline lipid NPs. HA enhanced the specific targeting of cubosomes with drug payloads to CD‐44‐expressing cancer cells.^[^ ^341^ ^]^ Quagliariello et al demonstrated the notable therapeutic efficacy of boronate‐based quercetin‐hyaluronic acid prodrug (HABQ) in prostate cancer therapy. HABQ treatment resulted in decreased hepatic level of inflammatory cytokines such as IL‐1 and IL‐6, and decreased level of protumor cytokines such as IL‐1b, IL‐6, and IL‐8 in cancer tissue, indicating both anti‐inflammatory and tumor‐inhibiting properties.^[^ ^342^ ^]^ Almeida et al developed an undecylenic acid‐modified thermally hydrocarbonized porous silicon nanocarrier covalently conjugated to a synthesized amide‐modified HA‐derived polymer (UnTHCPSi‐HA^+^) to target breast cancer. UnTHCPSi–HA^+^ demonstrated preferential accumulation in vivo due to the affinity of conjugated HA^+^ for CD44‐overexpressing tumors compared to non‐malignant tissues.^[^ ^343^ ^]^ HA has also been utilized for treating infectious diseases and inflammation. Yang et al. demonstrated that conjugation of oxidized HA can significantly improve serum stability of IFN‐α and its accumulation in the liver due to strong binding to IFN‐α receptors on the surface of liver sinusoidal epithelial cells.^[^ ^52^ ^]^ Upon intravenous injection, HA‐IFN‐α conjugate induced robust innate immune responses with the elevated expression of 2′‐5′‐oligoadenylate synthetase 1 in the virus‐infected liver. Recently, Lee et al. demonstrated the treatment of inflammatory bowel diseases using hyaluronic acid–bilirubin nanomedicine (HABN), which was formed by chemical conjugation and spontaneous nano‐aggregation in aqueous solution.^[^ ^53^ ^]^ HABN accumulated in the inflamed colonic epithelium via CD44‐mediated macrophage targeting and restored the intestinal barrier functions by the ROS‐scavenging effect of bilirubin in a murine dextran sulfate sodium‐induced colitis model. Importantly, HABN therapy was also associated with gut microbiota modulation, increasing its richness and diversity in favor of exerting strong anti‐inflammatory effects against acute colitis (**Figure** **8**a–e). The HA‐based hydrogel scaffold has been used in many preclinical and clinical studies by exploiting its capability of efficient loading and sustained release of a range of bioactive and immunologic agents, which allows localized recruitment and activation of immune cells for their robust and durable immunological activity.^[^ ^344^, ^345^ ^]^ Marino et al. developed a hyaluronic acid‐based, polyethylene glycol cross‐linked 3D dermal filler, called neauvia hydrogel, to modulate immunological functions of human polymorphonuclear leukocytes (PMN).^[^ ^346^ ^]^ Neauvia hydrogel treatment reduced migration, ROS production, and proinflammatory cytokine secretion of PMNs stimulated with N‐formylmethionyl‐leucyl‐phenylalanine, effectively regulating inflammatory responses. In another study, polyelectrolyte multilayer film was prepared by the layer‐by‐layer assembly of poly‐L‐lysine and HA‐aldehyde via charge interaction and covalent imine bonds.^[^ ^347^ ^]^ Polyelectrolyte multilayer film promoted the release of anti‐inflammatory cytokines, such as IL1‐RA and CCL18, while decreasing pro‐inflammatory cytokines of IL‐12, TNF‐α, and IL‐1β, resulting in monocyte differentiation to anti‐inflammatory M2 macrophages. Hu et al. reported an immunomodulatory alginate microcapsule that can inhibit TLR2‐mediated proinflammatory responses to improve islet transplantation therapy.^[^ ^348^ ^]^ In this study, TLR2‐blocking pectin polymer was engineered with methyl‐esterification for optimal performance, followed by pectin/alginate microcapsule formulation via Ca^2+^‐carboxylic acid coordination. It was shown that pectin/alginate microcapsules effectively reduced DAMP‐induced, TLR‐dependent NF‐κB activation in vitro and mitigated foreign body reactions and pro‐inflammatory cytokine/tissue responses in vivo. Accordingly, pectin/alginate microcapsules resulted in long‐term survival of encapsulated rat islets after xenotransplantation, leading to long‐term normoglycemia and superior glucose‐metabolism in diabetic mice.

### 5.5 Chitin and Chitosan

Chitin is a linear polysaccharide composed of N‐acetylglucosamine residues linked by beta‐(1,4)‐glycosidic bonds, and chitosan is its deacetylated derivative form with varied molecular weights and degrees of deacetylation. Chitin is the second most abundant polysaccharide in nature, constituting a major portion of the cell wall of fungi, exoskeletons of arthropods, and beaks of cephalopods, among many others. Chitin derivatives have shown a wide range of immune‐modulating effects that share many similarities with glucans, as N‐acetylglucosamine is a common main component of these polysaccharides.^[^ ^349^ ^]^ Immune recognition of chitin involves TLR‐2 signaling for IL‐12 and TNF‐α production and/or Dectin‐1 signaling for respiratory burst and phagocytosis. Chitin is sensed primarily in the lungs and gut by innate immune cells (e.g., eosinophils and macrophages) and Th2 cells that induce cytokine production, leukocyte recruitment, and alternative macrophage activation.^[^ ^350^ ^]^ Mami et al. prepared chitin microparticles via milling and sonication of chitin powder and investigated protective effects against Leishmania infection in mice. Subcutaneous injection of soluble Lishmania antigen (and chitin microparticles produced serum IgG against Lishmania antigen and significantly decreased lesion formation and parasite load levels of Leishmania major promastigotes with increased production of IFN‐γ, TNF‐α, and IL‐10 cytokines.^[^ ^351^, ^352^ ^]^

Chitosan has been extensively employed for encapsulation, protection, and pH‐dependent release of drugs and immunological agents. Wu et al. developed hydrophilic cationic polymer (N,N,N‐trimethyl chitosan, TMC)‐based nanocomplexes for the co‐delivery of DOX and rhIL‐2. The nanocomplex was prepared by covalently conjugating DOX to TMC through pH‐responsive cis‐aconitic anhydride, followed by electrostatic adsorption of rhIL‐2, which significantly delayed tumor growth with the increased levels of serum IgG and tumor‐infiltrated cytotoxic T lymphocytes.^[^ ^353^ ^]^ Song et al. synthesized an erythrocyte membrane‐coated nanogel to co‐deliver paclitaxel (PTX) and immune‐stimulating cytokine IL‐2 to the tumor. Hydroxypropyl‐B‐cyclodextrin acrylate and two oppositely charged chitosan derivatives (amphoteric methacrylamide N‐carboxyethyl chitosan and positively charged methacrylamide N‐(2‐hydroxy)propyl‐3‐trymethylammonium chitosan chloride) were used to entrap PTX and control pH‐responsive capability, respectively, while the red blood cell membrane was used to enhance the stability and extend systemic circulation for high tumor accumulation via the enhanced permeability and retention effect. The slightly acidic tumor microenvironment allowed sustained release of PTX, membrane disintegration, and constant release of IL‐2 for the stimulation of DCs, cytotoxic T‐lymphocytes, and NK cells for superior antitumor efficacy.^[^ ^354^ ^]^ Koppolu and Zaharoff prepared antigen‐encapsulated chitosan particles using OVA as a model protein antigen and showed that chitosan particles induced maturation and cytokine production of BMDCs and macrophages to stimulate activation and proliferation of antigen‐specific CD4^+^ and CD8^+^ T cells.^[^ ^355^ ^]^ Rebbouh et al. developed an anti‐toxin vaccine by loading chitosan NPs with Aah II toxin derived from Androctonus australis hector venom for protection against scorpion envenomation. The nanovaccine system induced blood neutrophilia and the associated myeloperoxidase activation and produced specific neutralizing antibodies for complete protection against lethal toxin challenge.^[^ ^356^ ^]^ Castro et al. prepared chitosan NPs through a coacervation method with poly(γ‐glutamic acid) adjuvant and showed that the NPs promoted the expression of co‐stimulatory molecules (i.e., CD86, CD40, and MHC‐II molecule) and the secretion of pro‐inflammatory cytokines (i.e., TNF‐α, IL‐12p40, and IL‐6) by human monocyte‐derived DCs and macrophages. They significantly increased CD8^+^ T cell proliferation and IFN‐γ secretion and reduced infiltration of M2‐like macrophages into RKO colorectal cancer.^[^ ^357^ ^]^ El‐Sissi et al. prepared Rift Valley Fever Virus‐chitosan NP vaccine using the ionic gelation method and loading with inactivated antigen. The vaccine NP upregulated gene expression of IL‐2, IFN‐γ, and IL‐4 associated with type‐1 and type‐2 immune responses, and significantly enhanced macrophage activity, neutralizing antibody titer, and total anti‐viral IgG levels.^[^ ^39^ ^]^ Shu et al developed a chitosan particle stabilized Pickering emulsion with IL‐12 (CSPE/IL12) as an adjuvant delivery system for plasmid gene protein 3 (Pgp3) protein due to the inefficient immune response by *C.trachomatis* plasmid encoded Pgp3 alone. In mice models infected by *Chlamydia muridarum, the* CSPE/IL‐12 vaccination regimen resulted in high levels of Pgp3‐specific antibodies, such as IgG, IgG1, IgG2a, and sIgA, and production of cytokines such as IFN‐γ, IL‐2, TNF‐α, and IL‐4, along with Th1 polarization, indicating elevated humoral and cellular immunity.^[^ ^358^ ^]^ Zhao et al synthesized a chitosan nanovaccine as a delivery adjuvant system for IL‐12 expression plasmid (Ng(‐)pIL‐12) to assess its adjuvant effect as a prophylactic hepatitis B virus (HBV) vaccine against pAAV/HBV1.2 challenge. Ng(‐)pIL‐12 adjuvant enhanced DCs maturation and antigen presentation. Additionally, it elicited strong HBV‐specific CD8^+^ and CD4^+^ T cell responses and high levels of anti‐HBs IgG, IgG1, and IgG2b while also inducing long‐term memory responses against HBV infection.^[^ ^359^ ^]^

Functional groups on the chitosan backbone allow chemical modifications that can improve the immunological activity of chitosan. Jiang et al. reported that carboxymethylated chitosan (CMCS) could induce an anti‐tumor immune response against BEL‐7402 human hepatic cancer cells in vitro and lung metastasis in mice bearing both hepatoma‐22 and H22 murine tumors. CMCS activated peritoneal macrophages to promote phagocytosis of murine hepatocellular cancer cells in vivo, and orally administered CMCS significantly slowed the tumor growth in mice bearing BEL‐7402 and hepatoma 22 cells by down‐regulation of vascular endothelial growth factor (VEGF) and MMP‐9 in tumors.^[^ ^360^ ^]^ Liang et al. developed a hydrogel using the Schiff base reaction between CpG‐modified CMCS and oxidized mannan for cancer vaccine application based on the adjuvant activity of CpG and mannan. In a murine B16F10 melanoma model, gel vaccine facilitated the functional maturation of DCs and macrophages in the tumor‐draining lymph nodes, leading to T cell‐mediated immune responses with subcutaneous administrations.^[^ ^361^ ^]^ Yang et al. developed chitosan micelles by chemical modification of chitosan with stearic acid and D‐mannose, which promoted the engagement and activation of DCs and macrophages via the mannose receptor. After intradermal administration, the micelles promoted secretion of pro‐inflammatory cytokines (i.e., IFN‐γ, TNF‐α, IL‐4, IL‐6, IL‐12) and increased tumor infiltration of CD4^+^ and CD8^+^ T cells for robust antitumor immune responses.^[^ ^362^ ^]^

To target the primary site of infection, chitosan has been used as an adjuvant in intranasal influenza vaccines to directly immunize the respiratory mucosa. Sui et al. created soluble matrix protein 2 of the avian influenza virus H9N2 strain by deleting the hydrophobic transmembrane domain and intranasally administered it to mice together with chitosan. The survival rate, residual lung virus titer, body weight change, and serum antibody titer indicated that immunization with matrix protein 2 and chitosan protected 100%, 90%, and 30% of the mice against lethal challenge of the homologous H9N2 and heterologous H1N1 and H5N1 viruses, respectively.^[^ ^363^ ^]^ Sawaengsak et al. developed chitosan NPs loaded with a split product of inactivated virions of the H1N1 strain, mainly composed of hemagglutinin, as a mucosal vaccine. The influenza vaccine developed increased systemic and mucosal antibody responses, IFN‐γ‐secreting cells in the spleen, and reduced influenza morbidity with 100% protection against a lethal influenza virus challenge.^[^ ^364^ ^]^ Hung et al. developed glucan‐chitin particles for vaccination against a multivalent polypeptide antigen that can induce large repertoires of B‐cell and T‐cell responses against fungal infection. Intranasal administration of the vaccine reduced fungal burdens in the lungs of human leukocyte antigen‐DR4 transgenic mice with a great level of mixed Th1 and Th17 responses. The results also demonstrated that chitin may account for the Th17 immune response via Dectin‐2 signaling, while glucan is responsible for the Th1 immune response via Dectin‐1 signaling.^[^ ^365^ ^]^ Oral administration of vaccines has many challenges, including antigen degradation in the acidic GI tract and inefficient cellular uptake by immune cells due to mucus barriers. To overcome these challenges, Cao et al. utilized alginate and chitosan for antigen protection against acidic degradation and enhanced uptake by immune cells, respectively. Calcium phosphate NPs were encapsulated with OVA and coated with alginate and chitosan at an optimal ratio for sustained release in simulated intestinal and colonic fluids. The chitosan on the NPs allowed efficient uptake by intestinal epithelial cells and macrophages and upregulated costimulatory molecules on macrophages, which led to significantly higher levels of mucosal IgA and serum IgG than free OVA protein.^[^ ^366^ ^]^ Maeyama et al examined the synergistic effect of chitosan and Type I interferon as mucosal adjuvants in stimulating antibody responses via intranasal administration for purposes of cancer and hepatitis treatment. Co‐administration of IFN‐β and chitosan increased “specific serum IgG and IgA antibody responses, the mucosal IgA antibody response, and antitoxin titers”. Similarly, the same trend was reported with IFN‐α, indicating that chitosan and Type I IFNs have a synergistic adjuvant effect.^[^ ^367^ ^]^

### 5.6 Pectin

Pectin is a polysaccharide consisting of α‐1,4‐linked D‐galacturonic acid and α‐1,2‐L‐rhamnose units, as well as numerous sugar residues such as arabinose and galactose. In past years, there has been a surge of research interests in various pharmacological applications of pectin, especially as an immune modulatory, anti‐inflammatory, or antitumor agent.^[^ ^368^ ^]^ Busato investigated the immunomodulating effects of pectin from broccoli via oral administration in mice. Pectin did not have an impact on the concentrations of pro‐inflammatory mediators such as NO, IL‐12, and IL‐1β in both in vitro and in vivo, while increasing the concentration of an anti‐inflammatory cytokine IL‐10 in vivo, signifying the anti‐inflammatory properties of pectin. This pectin also led to increased macrophage activation and phagocytic activity.^[^ ^369^ ^]^

Many studies have assessed how side‐chain modification impacts the anti‐cancer effects of pectin compared to unmodified pectin. For instance, a modified citrus pectin inhibited the carbohydrate‐binding protein called galectin‐3 with a subsequent inhibition of galectin‐3‐mediated functions in tumor growth, angiogenesis, and spontaneous metastasis in mice.^[^ ^370^ ^]^ The modified citrus pectin also induced a significant increase in the activation of human NK cells and lymphocytes, which improved the anticancer effect against chronic myeloid leukemia cells.^[^ ^371^ ^]^ Other sugar residues of the polymer, such as galactan and arabinose, were also found to contain structural elements that can bind to and block galectin‐3, whose expression is up‐regulated in many progressed cancers.^[^ ^372^ ^]^ Yang et al. investigated the anti‐cancer effects of rice hull polysaccharides (containing type II arabinogalactan. RHPS induced the expression of TNF‐related apoptosis‐inducing ligand, Fas ligand, perforin, and granzyme on NK‐cells and the secretion of IFN‐γ and TNF‐α upon intraperitoneal injection, which exerted cytotoxicity against CT26 colon cancer cells.^[^ ^373^ ^]^ Natarajamurthy et al. studied the effect of pectic polysaccharide (PP) sourced from swallow root (SRPP), carrot (CRPP), and ginger (GRPP) on GAL3 and GAL3 binding proteins (G3BP), which are implicated as key materials in developing the tumor metastasis of B16F10 cells. Incubation of B16F10 cells with SRPP, CRPP, and GRPP resulted in reduced expression of galectin‐3 and G3BP by 35‐60%. Additionally, retarded tumor cell growth indicated the antiproliferative effects of the plant‐based PPs and induction of apoptosis in association with the metastasis‐associated galectin‐3 and G3BP proteins.^[^ ^374^ ^]^

Angiogenesis is essential for the early‐stage growth of primary and metastatic tumors, which requires endothelial cell growth regulated by VEGF and matrix metalloproteinases such as MMP‐9.^[^ ^375^, ^376^ ^]^ Park et al. obtained pressured liquid extraction II of pectic polysaccharide from Persimmon leaves and investigated its effect on angiogenesis and MMP‐9 expression. Liquid extraction II treatment strongly downregulated VEGF and MMP‐9 mRNA expression and blocked the phosphorylation of signaling proteins relevant to angiogenesis (e.g., PI3K, AKT, p38, c‐Jun N‐terminal kinase, and p65) in human umbilical vein endothelial cells, which suggested anti‐angiogenic and anti‐metastatic properties of PLE‐II for the inhibition of tumor growth and metastasis.^[^ ^377^ ^]^ Arabinogalactan consists of galactan and arabinose immunomodulators that can activate immune cells and influence their surface protein expression levels to suppress tumor growth and metastasis. Lai et al. isolated type II arabinogalactan from *Anonectochilius formosanus*, which increased the expression of costimulatory molecules (i.e., CD86, CD83, CD80, CD40, MHC class I and II) in DC2.4 cells via TLR2 and TLR4.^[^ ^378^ ^]^ Similarly, Pandey et al. evaluated an arabinogalactan polysaccharide isolated from *Tinospora cordifolia* for the activation of BMDCs. The arabinogalactan polysaccharide induced the secretion of TNF‐α and IL‐12 to a greater extent than the lipopolysaccharide‐positive control group and promoted cross‐presentation and MHC‐II expression by BMDCs, leading to strong priming and cytotoxic activity of tumor‐specific cytotoxic T lymphocytes against a murine lymphoma model.^[^ ^379^, ^380^ ^]^

## 6 Future Perspective

The field of immunomodulation, encompassing immunoadjuvants, has become an area of focus in scientific investigation due to its remarkable potential and contribution to both basic research and clinical therapy.^[^ ^381^ ^]^ Notably, polysaccharides are gaining attention as promising novel adjuvants. The development of novel adjuvants relies on rational design to ensure optimal presentation of appropriate antigen with the right co‐stimuli in a suitable conformation, amount, and time for targeting specific cell populations. This necessitates a profound understanding of desired immune response against the pathogens, relevant immune cells and their localization, and expression of relevant PRRs. Progress in this knowledge is heavily dependent on animal models, particularly mice.^[^ ^382^ ^]^

Additionally, the understanding of key factors influencing polysaccharide‐PRR interaction sheds light on crucial aspects that can guide the development of future immunotherapeutic strategies. In a study comparing two CLR homologs, human and murine langerin, to evaluate the conservation of carbohydrate recognition, it was observed that the arrangement of receptors in polysaccharides is pivotal in modulating polysaccharide PRR interaction. While simple ligands exhibited nearly identical affinities and binding modes, complex polysaccharides displayed markedly different avidities and specificities between the two homologs. This highlights the importance of the arrangement of receptors in the polysaccharides and the overall structure of the polysaccharide, rather than the mere presence of simple molecular patterns or glycans. While carbohydrate monomer binding is weak, multivalent display of both glycan and receptor significantly increases binding avidities, affinity, and specificity.^[^ ^383^, ^384^, ^385^ ^]^ Evidently, minor differences in or around the CRD site, including variations in residues neighboring the binding site, also influence ligand specificity. Differences in secondary binding sites despite sharing primary binding sites may also affect the ligand specificity for complex polysaccharides.^[^ ^384^ ^]^ In particular, particulate polysaccharides demonstrated that multiple receptor interactions with several heterogeneous PRRs resulted in amplified signaling.^[^ ^237^, ^384^ ^]^ Particulate polysaccharides also strongly influence PAMP‐PRR signaling through surface ligand arrangement.^[^ ^237^ ^]^ Thus, exploring the particulate form of polysaccharides holds promise in exploiting the engineered benefits of PAMP‐PRR interactions.

## Conflict of Interest

J.J.M declares financial interests as board membership, a paid consultant, research funding, and/or equity holder in EVOQ Therapeutics and Saros Therapeutics. The University of Michigan has a financial interest in EVOQ Therapeutics.
